# Association of circulating vitamin levels with thyroid diseases: a Mendelian randomization study

**DOI:** 10.3389/fendo.2024.1360851

**Published:** 2024-06-11

**Authors:** Wenke Zhang, Erhao Liu, Huafa Que

**Affiliations:** ^1^ Department of Traditional Chinese Surgery, Longhua Hospital Shanghai University of Traditional Chinese Medicine, Shanghai, China; ^2^ Longhua Medical College, Shanghai University of Traditional Chinese Medicine, Shanghai, China

**Keywords:** vitamins, autoimmune hyperthyroidism, autoimmune hypothyroidism, thyroid nodules, thyroid cancer, Mendelian randomization, causal effect

## Abstract

**Background:**

Previous observational studies have shown conflicting results of vitamins supplementation for thyroid diseases. The causal relationships between vitamins and thyroid diseases are unclear. Therefore, we conducted a two-sample bidirectional Mendelian randomization (MR) study to explore association of circulating vitamin levels with thyroid diseases.

**Methods:**

We performed a bidirectional MR analysis using genome-wide association study (GWAS) data. Genetic tool variables for circulating vitamin levels include vitamins A, B_9_, B_12_, C, D, and E, Genetic tool variables of thyroid diseases include autoimmune hyperthyroidism, autoimmune hypothyroidism, thyroid nodules (TNs), and Thyroid cancer (TC). Inverse-variance weighted multiplicative random effects (IVW-RE) was mainly used for MR Analysis, weighted median (WM) and MR Egger were used as supplementary methods to evaluate the relationships between circulating vitamin levels and thyroid diseases. Sensitivity and pluripotency were evaluated by Cochran’s Q test, MR-PRESSO, Radial MR, MR-Egger regression and leave-one-out analysis.

**Results:**

Positive MR evidence suggested that circulating vitamin C level is a protective factor in autoimmune hypothyroidism (OR_IVW-RE_=0.69, 95%CI: 0.58-0.83, *p* = 1.05E-04). Reverse MR Evidence showed that genetic susceptibility to autoimmune hyperthyroidism is associated with reduced level of circulating vitamin A(OR_IVW-RE_ = 0.97, 95% CI: 0.95–1.00, *p* = 4.38E-02), genetic susceptibility of TNs was associated with an increased level of circulating vitamin D (OR_IVW-RE_ = 1.02, 95% CI: 1.00–1.03, *p* = 6.86E-03). No causal and reverse causal relationship was detected between other circulating vitamin levels and thyroid diseases.

**Conclusion:**

Our findings provide genetic evidence supporting a bi-directional causal relationship between circulating vitamin levels and thyroid diseases. These findings provide information for the clinical application of vitamins prevention and treatment of thyroid diseases.

## Introduction

Thyroid diseases include benign and malignant diseases of the thyroid and pose a significant public health risk. Among them, TC, Autoimmune thyroid disease (AITD) and TNs are the clinically common diseases. TC is a common endocrine cancer. According to statistics, 19.3 million new cancers and 10 million deaths occurred in 2020, worldwide. TC accounted for 3.0 % and 0.4 % respectively (1). Compared with other cancers, the overall prognosis of TC is advantageous, but the financial and psychological pressure it brings to patients can not be underestimated. AITD affects 2% to 5% of the general population and includes Graves disease (GD) and Hashimoto disease (HD), common causes of hyperthyroidism and hypothyroidism, respectively (2). The prevalence of TNs ranges from 4 to 67%. Most TNs are benign, but 5% to 10% of TNs have malignant signs (3). For thyroid diseases, early identification and active treatment are crucial. It is crucial to find new risk factors and possible causal relationships in terms of prevention and treatment.

Vitamins are essential trace elements in the pathophysiological process of thyroid. Vitamin A and its derivatives, by binding to its receptors, can affect thyroid hormone (TH) signal transduction (4), regulate the effect of TH on target tissues (5), and increase the binding rate of thyroid stimulating hormone (TSH) to thyroid cells (6). B vitamins are involved in the process of oxidative stress caused by chronic inflammation, which causes increased levels of homocysteine in the blood, and hyperhomocysteinemia (Hcy) is closely associated with hypothyroidism (7). The occurrence and development of thyroid-related diseases are closely related to REDOX imbalance. Vitamins C and E have a strong ability to regulate REDOX. Studies have shown that vitamins C and E can improve oxidative damage in patients with thyroid diseases (8, 9). In 1994, Berg discovered the expression of vitamin D receptors on thyroid follicular cells in rats, suggesting that vitamin D may be involved in the pathophysiological processes of the thyroid (10). Experimental studies in rats suggested that vitamin D may have central and peripheral effects on the release of TSH and TH. Currently, clinical trials of vitamin D supplementation for AITD and TC have been conducted, but the results have been inconsistent (11).

In order to further uncover the correlation between circulating vitamin levels and thyroid diseases, more rigorous studies are needed. However, traditional observational studies are often affected by confounding factors and reverse causality, and there are certain limitations in the reliability of results. Therefore, we conducted an MR study of multiple circulating vitamin levels(vitamin A, B_9_, B_12_, C, D, and E) and thyroid diseases(autoimmune hyperthyroidism, autoimmune hypothyroidism, TNs, and TC). Unlike traditional studies, MR studies genetically explain the cause-and-effect relationships between circulating vitamin levels and thyroid diseases. In MR studies, because parents’ alleles are randomly assigned at conception, genetic variation precedes disease development and is not influenced by environmental confounders. Therefore, MR studies avoid confounding bias and reverse causality, and the research results are more robust and reliable (12).

## Materials and methods

### Study design

Since the publicly available databases we use have been approved by their respective ethics review committees. Therefore, this study does not require ethical approval.

We studied the causal relationship between circulating vitamin levels and thyroid diseases using bidirectional two-sample MR. Vitamin A, B_9_, B_12_, C, D and E were included in this study. The types of thyroid diseases were autoimmune hyperthyroidism, autoimmune hypothyroidism, TNs, and TC.

MR research must conform to three assumptions: 1) Correlation hypothesis: The genetic variation selected as an instrumental variables (IVs) must be strongly correlated with exposure. 2) Independence hypothesis: Genetic variation is not associated with anything that might confuse expose-outcome causality. 3) Exclusion of the limiting hypothesis: IVs affect results only through exposure.

### Data source

To minimize heterogeneity, we only used data from the European Population Bank. We obtained GWAS summary statistics for vitamin A (13) and B_9_ (14) from a publicly available database of the GWAS Catalog, with sample sizes of 8,247 and 5998, respectively. The UK Biobank project is a prospective cohort study with deep genetic and phenotypic data. A rich variety of phenotypic and health-related information was provided by approximately 500,000 people, including lifestyle indicators, biomarkers in blood and urine, and imaging of the body and brain. The GWAS summary statistics of vitamin B_12_, C, D, and E were from the UK Biobank, with a sample size of 64,979.

The FinnGen research project involves collecting and analyzing genomic data from 500,000 Finnish biobank participants in order to identify genetic variants associated with various health conditions. The summary statistics of thyroid diseases were all from the FinnGen (r9.finngen.fi), Including autoimmune hyperthyroidism (1828 cases of autoimmune hyperthyroidism and 279,855 control cases), autoimmune hypothyroidism (40,926 cases of autoimmune hypothyroidism and 274,069 control cases), and TNs (9485 nontoxic goiter/thyroid nodule and 367792 control group), TC (1783 malignant neoplasm of thyroid gland and 287,137 controls). Details of the phenotypes are shown in **Table 1**.

**Table 1:** Data source and detailed information of circulating vitamin levels and thyroid diseases.

Traits	Data sources(ID)	Sample size	Ncases	Ncontrols	Ancestry
Vitamin A	GWAS Catalog (GCST90200405)	8247	/	/	European
Vitamin B_9_	GWAS Catalog (GCST90012742)	5998	/	/	European
Vitamin B_12_	United Kingdom Biobank (ukb-b-19524)	64979	/	/	European
Vitamin C	United Kingdom Biobank (ukb-b-19390)	64979	/	/	European
Vitamin D	United Kingdom Biobank (ukb-b-18593)	64979	/	/	European
Vitamin E	United Kingdom Biobank (ukb-b-6888)	64979	/	/	European
Autoimmune hyperthyroidism	FinnGen(finngen_R9_AUTOIMMUNE_HYPERTHYROIDISM)	281683	1828	279855	European
Autoimmune hypothyroidism	FinnGen(finngen_R9_E4_HYTHY_AI_STRICT)	314995	40926	274069	European
Nontoxic goitre/Thyroid nodule	FinnGen(finngen_R9_E4_NONTOXIC_THYROID)	377277	9485	367792	European
Malignant neoplasm of thyroid gland	FinnGen(finngen_R9_C3_THYROID_GLAND_EXALLC)	288920	1783	287137	European

### Selection of IVs

We used TwoSampleMR (version 0.5.8) in the R package (version 4.3.2) to select SNPs (single nucleotide polymorphism) that fit the above three hypotheses. First, there were enough SNPs for MR analysis. We selected the IVs that are associated with exposure (*p* < 5E-06) (15). Second, to ensure that each SNP is independent, we clumped the data (r2 = 0.001, clumping window = 10,000 kb). These SNPs were extracted from the resulting GWAS, and we did nothing with the missing SNPs. We deleted palindromic SNPs and incompatible SNPs. Finally, we evaluated the statistical power of each IV by calculating the F value (F = beta^2^/se^2^) (16) and eliminated weak IVs with F<10.

### Statistical analysis of MR

Three methods, IVW-RE, WM, and MR Egger (17) were selected for MR Analysis. To reduce the variation heterogeneity and pleiotropic effect. IVW-RE analysis was the main result, and p<0.05 indicated that there was a causal relationship between exposure and outcome. Selected SNPs needed to be examined by heterogeneity and sensitivity analysis to ensure the robustness of the MR Analysis results. The Cochrane Q test (18), which included the MR-egger method and inverse variance weighted method, was used to assess heterogeneity. The MR-Egger intercept (19) was also performed to evaluate horizontal pleiotropy. At the same time, MR-PRESSO packages (20) and Radial MR packages (21) are used to detect SNPs with heterogeneity and remove these SNPs in the final analysis. In addition, the leave-one-out method (22) is used as a sensitivity analysis, excluding one SNP at a time and performing an IVW on the remaining SNPs to detect the potential impact of SNPs with high pleiotropy levels on MR results.

## Result

### Causal effects of circulating vitamin levels on autoimmune hyperthyroidism

**Figure 1:**
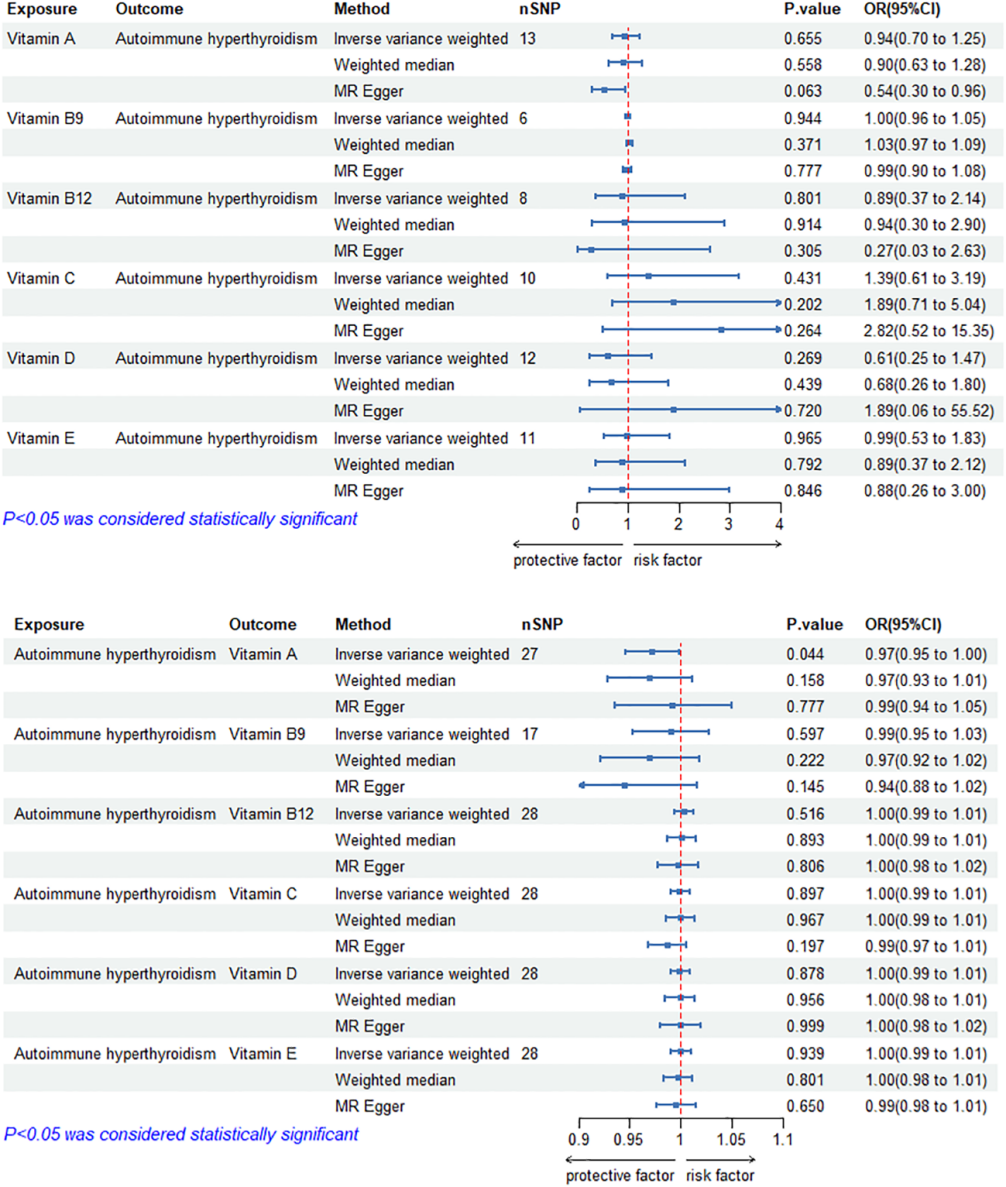
Bidirectional causal estimation of circulating vitamin levels and autoimmune hyperthyroidism. nSNPs, the number of SNPs used in MR; OR, odds ratio; CI, confidence interval.

The MR results of circulating vitamin levels and autoimmune hyperthyroidism are shown in **Figure 1**. There was no weak tool bias (F>10) for any IV used in the analysis (**Supplementary Material 2**).

In reverse MR analysis, genetically predicted autoimmune hyperthyroidism showed a negative causal relationship with circulating vitamin A level (OR_IVW-RE_= 0.97, 95% CI: 0.95–1.00, *p* =4.38E-02). In addition, we did not find a significant causal relationship between circulating vitamin B_9_, B_12_, C, D and E levels and autoimmune hyperthyroidism (*p*>0.05). Cochran’s Q test and the MR-Egger intercept confirmed that there was no heterogeneity and pleiotropy in our study (**Table 1**, **Supplementary Material 1**). The application of the leave-one-out method improved the reliability of MR analysis results (**Supplementary Material 3**).

### Causal effects of circulating vitamin levels on autoimmune hypothyroidism

**Figure 2:**
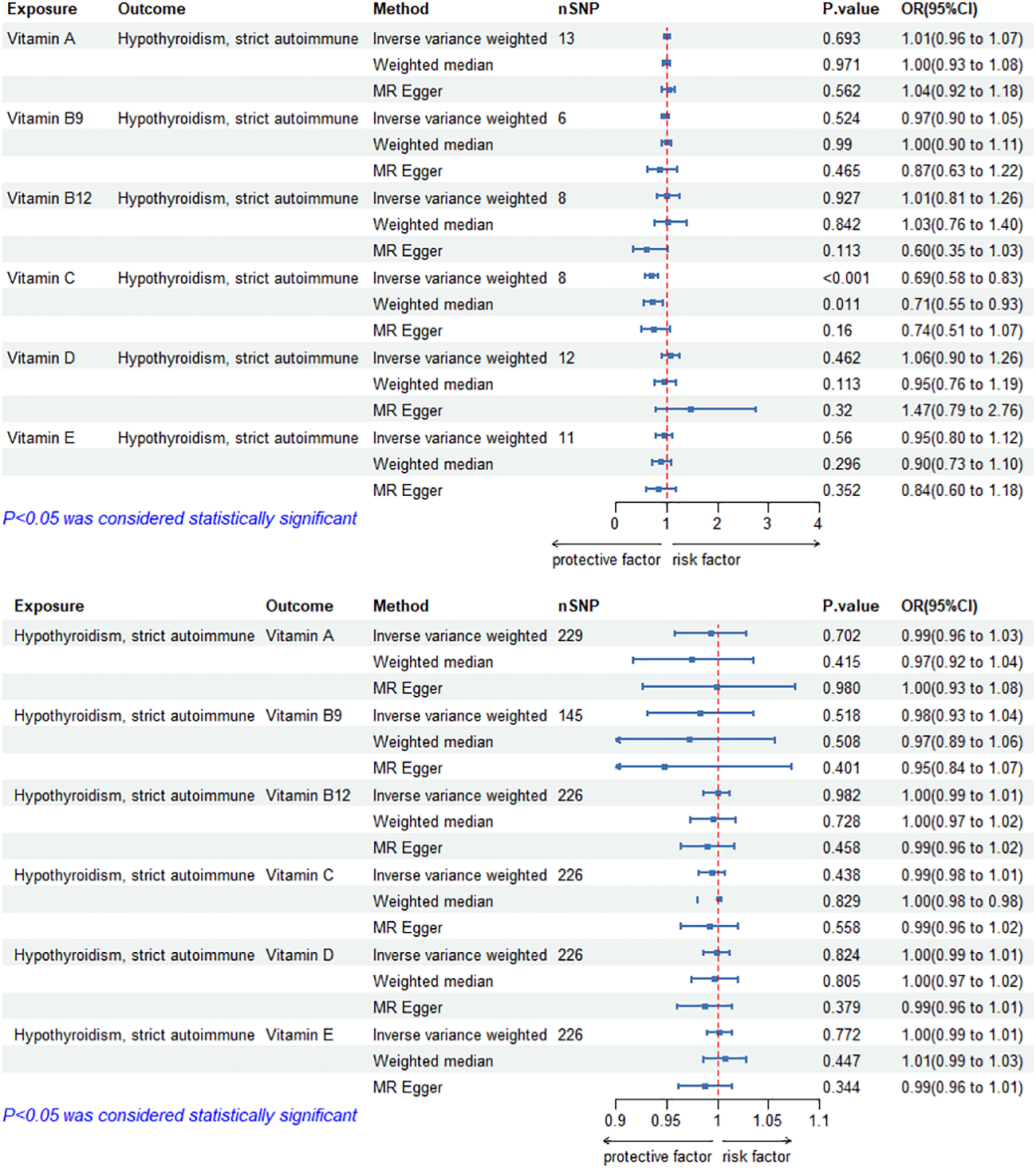
Bidirectional causal estimation of circulating vitamin levels and autoimmune hypothyroidism. nSNPs, the number of SNPs used in MR; OR, odds ratio; CI, confidence interval.

The MR results of circulating vitamin levels and autoimmune hypothyroidism are shown in **Figure 2**. There was no weak tool bias (F>10) for any IV used in the analysis (**Supplementary Material 2**).

In MR Analysis, the IVW-RE method showed no causal relationship between circulating vitamin C level and autoimmune hypothyroidism (*p*>0.05). However, Cochran’s Q test showed heterogeneity (*p*<0.05). After the removal of heterogeneous SNPs (rs1883993, rs11650824) through MR-PRESSO and Radial MR analysis, the result showed a causal relationship between circulating vitamin C level and autoimmune hypothyroidism. circulating vitamin C has a protective effect on autoimmune hypothyroidism (OR_IVW-RE_=0.69, 95%CI: 0.58-0.83, *p* = 1.05E-04). WM methods reached the same conclusion (OR_WM_=0.71, 95%CI: 0.55-0.93, *p* = 1.13E-02). There was no heterogeneity (*p*>0.05) or pleiotropy (*p*>0.05). Except for vitamin C, we found no association between other vitamins and autoimmune hypothyroidism. Cochran’s Q test and the MR-Egger intercept confirmed that there was no heterogeneity and pleiotropy in our study (**Table 2**, **Supplementary Material 1**). The application of the leave-one-out method improved the reliability of MR analysis results (**Supplementary Material 3**).

### Causal effects of circulating vitamin levels on TNs

**Figure 3:**
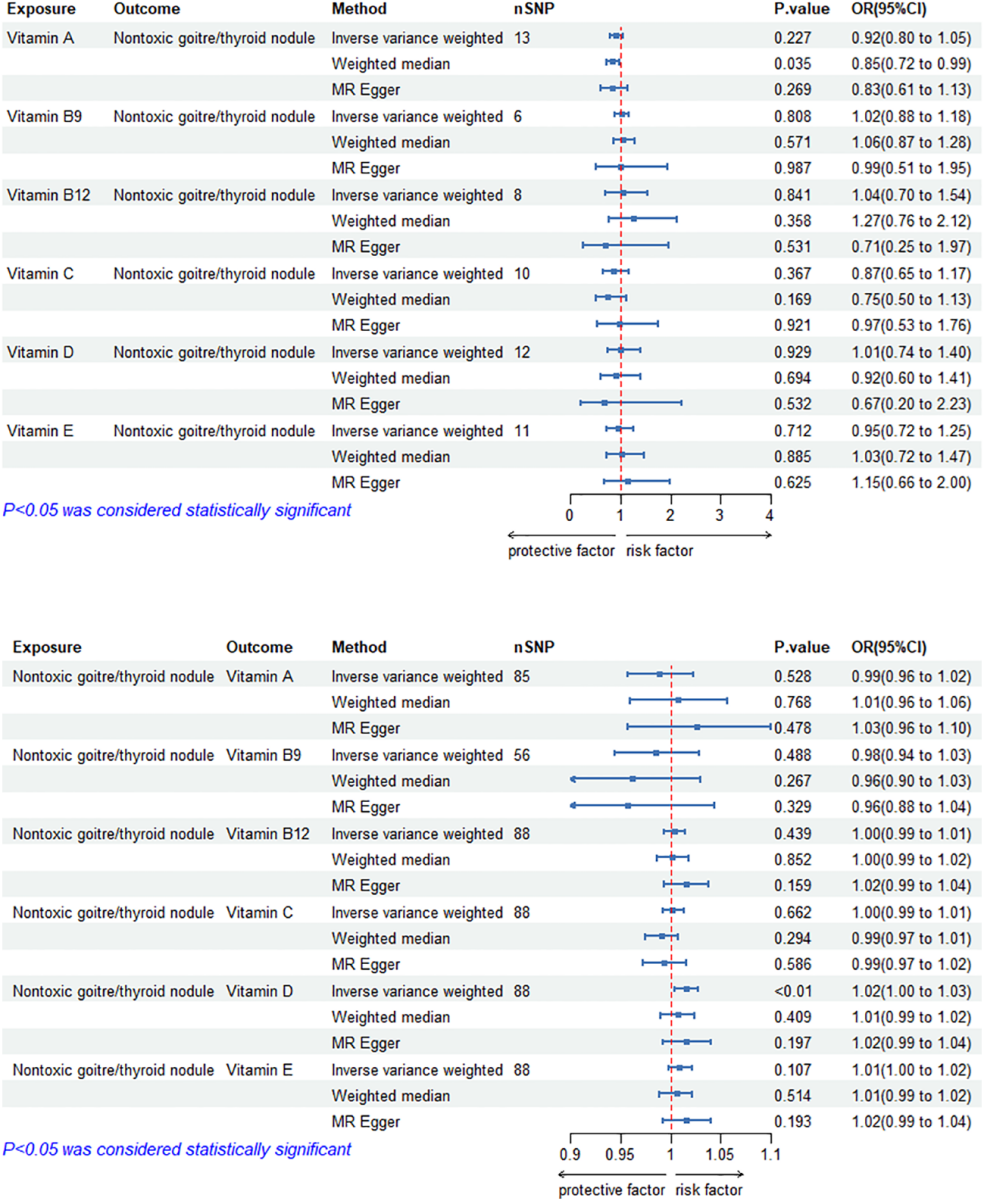
Bidirectional causal estimation of circulating vitamin levels and TNs. nSNPs, the number of SNPs used in MR; OR, odds ratio; CI, confidence interval.

The MR results of circulating vitamin levels and TNs are shown in **Figure 3**. There was no weak tool bias (F>10) for any IV used in the analysis (**Supplementary Material 2**).

In reverse MR analysis, The causal relationship between genetically predicted TNs and circulating vitamin D level was positive (OR_IVW-RE_= 1.02, 95% CI: 1.00–1.03, *p* = 6.86E-03). Besides, we did not find a significant causal relationship between circulating vitamin A, B_9_, B_12_, C and E levels and TNs (*p*>0.05). Cochran’s Q test and the MR-Egger intercept confirmed that there was no heterogeneity and pleiotropy in our study (**Table 3**, **Supplementary Material 1**). The application of the leave-one-out method improved the reliability of MR analysis results(**Supplementary Material 3**).

### Causal effects of circulating vitamin levels on TC

**Figure 4:**
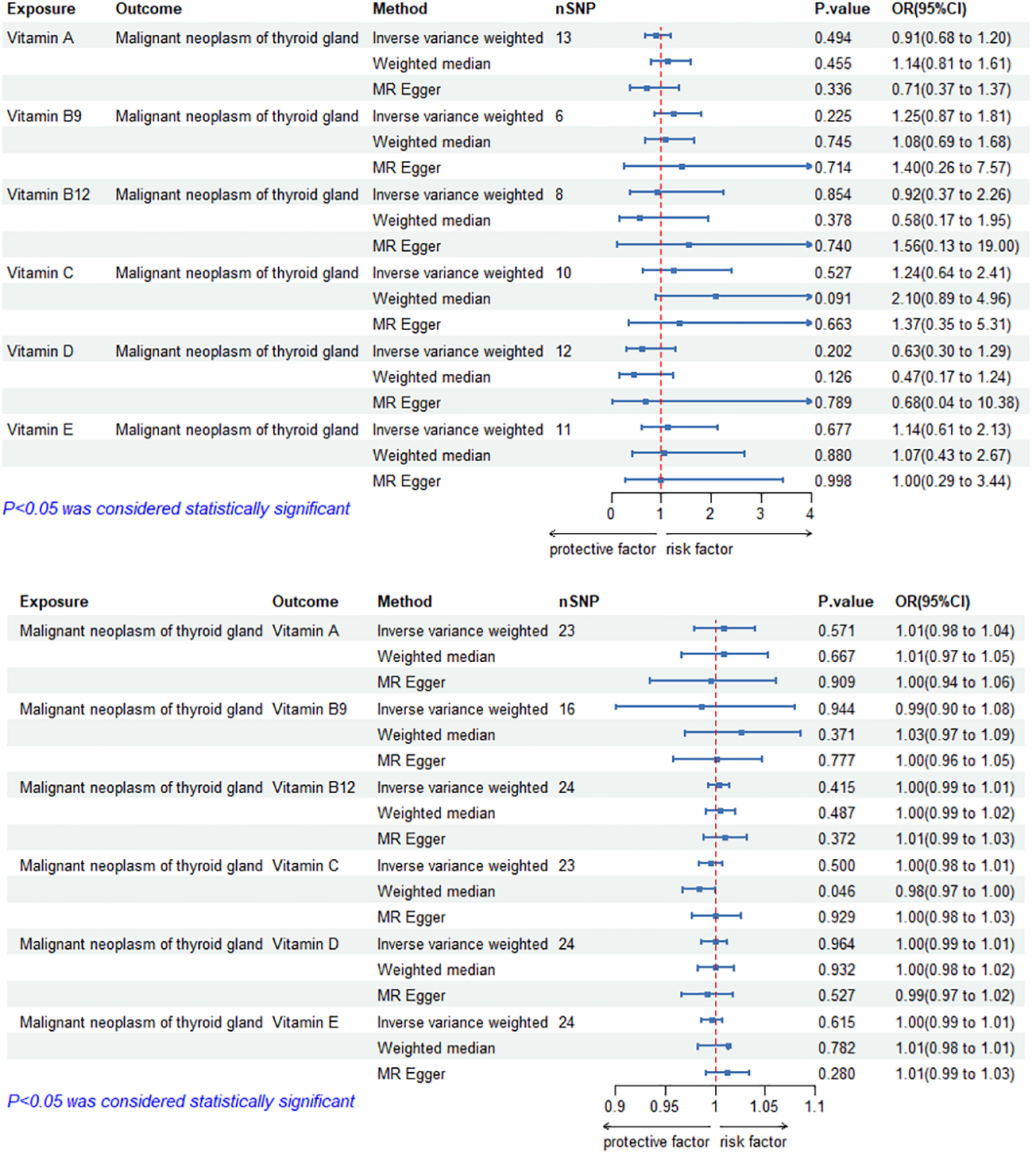
Bidirectional causal estimation of circulating vitamin levels and TC. nSNPs, the number of SNPs used in MR; OR, odds ratio; CI, confidence interval.

The MR results of circulating vitamin levels and TC are shown in **Figure 4**. There was no weak tool bias (F>10) for any IV used in the analysis (**Supplementary Material 2**).

In MR analysis, the IVW-RE method showed no causal relationship between circulating vitamin levels and TC (*p*>0.05). The results of The MR Egger and WM methods were consistent with those of the IVW-RE method(*p*>0.05). After the removal of outliers by the MR-PRESSO and Radial MR During the MR Analysis, it was suggested that heterogeneity and pleiotropy disappeared after secondary testing. (**Table 4**, **Supplementary Material 1**). The application of the leave-one-out method improved the reliability of MR analysis results(**Supplementary Material 3**).

## Discussion

In this study, we performed the bidirectional causal relationship between circulating vitamin levels and thyroid diseases using a bidirectional two-sample MR Study. Genetic evidence suggested that circulating vitamin C level has a protective effect on autoimmune hypothyroidism. In reverse MR, The results showed that reduced vitamin A levels are related to the risk of developing autoimmune hyperthyroidism, and vitamin D increase can contribute to development of TNs. In addition, there was no significant association between circulating vitamin levels and thyroid diseases.

Vitamin C is a member of the body’s non-enzymatic antioxidant system. It is a cofactor of many biosynthetic and gene regulatory enzymes, and plays an important role in a variety of immune regulation, chromatin remodeling, and cell division (23). At the physiological concentration of human plasma vitamin C (40-80μM), it can effectively remove reactive oxygen species (ROS) and act as an antioxidant; at high doses (10-20 mM), vitamin C can induce oxidative stress and inhibit tumor growth without significant damage to normal cells and tissues (23). An animal study demonstrated a protective effect of vitamin C against boldenone undecylenate-induced autoimmune hypothyroidism of Wistar rats (24). Karimi F (25) randomly divided 100 patients with autoimmune thyroiditis into the Selenium (Se) treatment group, vitamin C treatment group, and placebo treatment group. antithyroid peroxidase antibody (TPO-Ab) concentration decreased in both Se and vitamin C treated groups, but no change was observed in the placebo group. The above studies are consistent with the results of this study. The pathogenesis of autoimmune hypothyroidism is related to oxidative stress (26) and abnormal immune system (27). Vitamin C may affect gene expression of oxidative stress and immune system, and has a protective effect on autoimmune hypothyroidism. Although evidence of large-scale evidence-based medicine is still lacking, this study provides new ideas for improving our understanding of the pathogenesis and treatment of autoimmune hypothyroidism.

Studies (28) have also shown that high doses of vitamin C can affect the REDOX equilibrium and cell metabolism of cancer cells by mediating the pro-oxidation mechanism, thereby inhibiting the growth of TC cells. However, this MR study did not observe a direct causal relationship between vitamin C and TC, which may be related to the small number of cases of TC, or may have an anti-tumor effect through indirect effects, but no matter which possibility, it is worth further study.

On the other hand, the results of this study showed that autoimmune hyperthyroidism is a risk factor for reduced vitamin A levels. Vitamin A is able to regulate thyroid homeostasis alone or interact with other micronutrients, especially with iodine (29). And vitamin A also has antioxidant effects, and the pathogenesis of autoimmune hyperthyroidism is related to impaired oxidative stress. The decrease in vitamin A level may be due to its participation in the process of regulating oxidative stress damage. A study has shown that vitamin A can alleviate the clinical symptoms of patients with hyperthyroidism and reduce the metabolic rate (30). More and more studies have also shown that the levels of vitamin A and its derivatives can regulate TH from gene expression levels and their effects on target tissues (31). However, whether vitamin A supplementation can make hyperthyroidism gain clinical benefits and possible channels of action are worth looking forward to in future large-scale randomized controlled studies.

Vitamin D_3_ is one of the most important types of vitamin D in the body. Calcitriol (the active form of vitamin D3) can bind to the vitamin D receptor (VDR). VDR is involved in regulating the expression of more than 1000 genes, including thyroid tissue (32–34). More and more studies have shown that vitamin D deficiency can cause thyroid dysfunction and autoimmune thyroid disease (11), and vitamin D supplementation can improve thyroid function and treat autoimmune thyroid disease (35, 36). However, vitamin D supplementation did not reduce the overall risk of hypothyroidism (37); It also had poor therapeutic effect on GD patients (38). Another study (39) suggested that vitamin D deficiency was a risk factor for TC and that vitamin D supplementation inhibited the proliferation (40) and ability of TC cells to metastasize (41) and reduced the risk of advanced cancer in individuals who were undiagnosed at the start of the study (42). However, a study (43) has shown no link between vitamin D levels and the risk of TC. The direct causal relationship between TC and autoimmune thyroid disease and vitamin D has not been observed in our MR study. This can also explain the contradiction of the current clinical research results. There were few studies on the relationship between TNs and vitamin D. Jinzhuo Fan found a negative correlation between TNs and vitamin D levels in a study of 875 centenarians in Hainan Province, China (44). A study in Turkey also found that the level of vitamin D was low in patients with TNs (45). This is the opposite of our study. The MR Study was independent of environmental confounding factors, but we included only European populations. Turkey straddles Asia and Europe, and China belongs to Asia. Therefore, the inconsistency of the results may be due to the different populations.Therefore, what is the optimal level of vitamin D maintenance in patients with thyroid diseases and can benefit from the protective effect of vitamin D needs to be further determined by large sample and multi-center clinical studies.

Vitamin E can play an antioxidant role by acting as a structural component of biofilms as well as cleaning up free radicals (46). But the role of vitamin E in thyroid diseases has not been fully appreciated. Only a few studies have shown that vitamin E reduces the aggressiveness of TC in patients (47) and reduces oxidative damage caused by excessive TH in the blood circulation of hyperthyroid animals (9). However, our MR Study did not show any statistically significant direct cause-and-effect relationship between vitamin E and thyroid diseases. The vitamin B family is involved in folate and homocysteine metabolism in different ways. Deficiencies in folic acid, vitamins B_9_, and B_12_ can cause increased levels of homocysteine in the blood (48, 49), but Hcy is associated with impaired TH sensitivity in adults with normal thyroid function (50). No studies have shown a direct association between vitamin B_9_ and B_12_ and thyroid diseases, which is consistent with our findings.

There are some limitations to this study. Firstly, although our inclusion of only European populations avoids the bias caused by racial stratification, it also limits the possibility of generalizing our study to the entire population. Secondly, to have sufficient IVs in MR Analysis, we weakened the independence of SNPs (*p* < 5E-06), but the F statistics of SNPs are all greater than 10, and they meet the conditions of MR Analysis. Thirdly, although we use the latest statistical data with the largest sample size, the small sample size still limits the reliability of MR analysis. Nevertheless, our study is the first to elucidate the association between circulating vitamins and thyroid diseases at the genetic level, providing new insights into the relationship between the two and providing valuable information for the study of the pathogenesis of thyroid diseases.

## Conclusion

Taken together, this study reveals a bidirectional causal relationship between circulating vitamin levels and thyroid diseases, providing new insights and evidence for the etiology, screening, and management of thyroid diseases and clinical micronutrient deficiencies. Further research is needed to elucidate the underlying mechanisms between vitamins and thyroid diseases and to verify this association through basic experiments as well as large-scale randomized controlled trials.

## Data availability statement

The original contributions presented in the study are included in the article/**Supplementary Material**. Further inquiries can be directed to the corresponding author.

## Author contributions

WZ: Data curation, Writing – original draft, Methodology. EL: Data curation, Visualization, Writing – original draft. HQ: Writing – review & editing, Conceptualization, Data curation, Methodology.

## References

[1] Sung H Ferlay J Siegel RL Laversanne M Soerjomataram I Jemal A. Global cancer statistics 2020: GLOBOCAN estimates of incidence and mortality worldwide for 36 cancers in 185 countries. CA Cancer J Clin. (2021) 71(3):209–49. doi: 10.3322/caac.21660 33538338

[2] Petranović Ovčariček P Görges R Giovanella L. Autoimmune thyroid diseases. Semin Nucl Med. (2024) 54(2):219–36. doi: 10.1053/j.semnuclmed.2023.11.002 38044176

[3] Gökmen Inan N Kocadağlı O Yıldırım D Meşe İ Kovan Ö. Multi-class classification of thyroid nodules from automatic segmented ultrasound images: Hybrid ResNet based UNet convolutional neural network approach. Comput Methods Programs Biomed. (2024) 243:107921. doi: 10.1016/j.cmpb.2023.107921 37950926

[4] Li H Bai B Zhang Q Bao Y Guo J Chen S. Ectopic cross-talk between thyroid and retinoic acid signaling: a possible etiology for spinal neural tube defects. Gene. (2015) 573:254–60. doi: 10.1016/j.gene.2015.07.048 26188161

[5] Froöhlich E Witke A Czarnocka B Wahl R. Retinol has specific effects on binding of thyrotrophin to cultured porcine thyrocytes. J Endocrinol. (2004) 183:617–26. doi: 10.1677/joe.1.05693 15590987

[6] Kogai T Liu Y-Y Richter LL Mody K Kagechika H Brent GA. Retinoic acid induces expression of the thyroid hormone transporter, monocarboxylate transporter 8 (Mct8). J Biol Chem. (2010) 285:27279–88. doi: 10.1074/jbc.M110.123158 PMC2930727 20573951

[7] Cicone F Santaguida MG My G Mancuso G Papa A Persechino R. Hyperhomocysteinemia in acute iatrogenic hypothyroidism: the relevance of thyroid autoimmunity. J Endocrinol Invest. (2018) 41:831–7. doi: 10.1007/s40618-017-0811-y 29288439

[8] Farasati Far B Behnoush AH Ghondaghsaz E Habibi MA Khalaji A. The interplay between vitamin C and thyroid. Endocrinol Diabetes Metab. (2023) 6:e432. doi: 10.1002/edm2.432 37246589 PMC10335618

[9] Napolitano G Fasciolo G Di Meo S Venditti P. Vitamin E supplementation and mitochondria in experimental and functional hyperthyroidism: A mini-review. Nutrients. (2019) 11:2900. doi: 10.3390/nu11122900 31805673 PMC6950234

[10] Berg JP Liane KM Bjørhovde SB Bjøro T Torjesen PA Haug E. Vitamin D receptor binding and biological effects of cholecalciferol analogues in rat thyroid cells. J Steroid Biochem Mol Biol. (1994) 50:145–50. doi: 10.1016/0960-0760(94)90021-3 8049143

[11] Babić Leko M Jureško I Rozić I Pleić N Gunjača I Zemunik T. Vitamin D and the thyroid: A critical review of the current evidence. Int J Mol Sci. (2023) 24:3586. doi: 10.3390/ijms24043586 36835005 PMC9964959

[12] Hemani G Zheng J Elsworth B Wade KH Haberland V Baird D. The MR-Base platform supports systematic causal inference across the human phenome. Elife. (2018) 7:e34408. doi: 10.7554/eLife.34408 29846171 PMC5976434

[13] Chen Y Lu T Pettersson-Kymmer U Stewart ID Butler-Laporte G Nakanishi T. Genomic atlas of the plasma metabolome prioritizes metabolites implicated in human diseases. Nat Genet. (2023) 55:44–53. doi: 10.1038/s41588-022-01270-1 36635386 PMC7614162

[14] Dennis JK Sealock JM Straub P Lee YH Hucks D Actkins K. Clinical laboratory test-wide association scan of polygenic scores identifies biomarkers of complex disease. Genome Med. (2021) 13:6. doi: 10.1186/s13073-020-00820-8 33441150 PMC7807864

[15] Zhong S Yang W Zhang Z Xie Y Pan L Ren J. Association between viral infections and glioma risk: a two-sample bidirectional Mendelian randomization analysis. BMC Med. (2023) 21:487. doi: 10.1186/s12916-023-03142-9 38053181 PMC10698979

[16] Bowden J Holmes MV. Meta-analysis and Mendelian randomization: A review. Res Synth Methods. (2019) 10:486–96. doi: 10.1002/jrsm.1346 PMC6973275 30861319

[17] Chen X Kong J Pan J Huang K Zhou W Diao X. Kidney damage causally affects the brain cortical structure: A Mendelian randomization study. EBioMedicine. (2021) 72:103592. doi: 10.1016/j.ebiom.2021.103592 34619639 PMC8498227

[18] Bowden J Del Greco MF Minelli C Davey Smith G Sheehan N Thompson J. A framework for the investigation of pleiotropy in two-sample summary data Mendelian randomization. Stat Med. (2017) 36:1783–802. doi: 10.1002/sim.7221 PMC5434863 28114746

[19] Bowden J Davey Smith G Burgess S. Mendelian randomization with invalid instruments: effect estimation and bias detection through Egger regression. Int J Epidemiol. (2015) 44:512–25. doi: 10.1093/ije/dyv080 PMC4469799 26050253

[20] Verbanck M Chen CY Neale B Do R. Detection of widespread horizontal pleiotropy in causal relationships inferred from Mendelian randomization between complex traits and diseases [published correction appears in Nat Genet. Nat Genet. (2018) 50:693–8. doi: 10.1038/s41588-018-0099-7 PMC6083837 29686387

[21] Bowden J Spiller W Del Greco MF Sheehan N Thompson J Minelli C. Improving the visualization, interpretation and analysis of two-sample summary data Mendelian randomization via the Radial plot and Radial regression. Int J Epidemiol. (2018) 47:2100. doi: 10.1093/ije/dyy265 30423109 PMC6280936

[22] Bowden J Del Greco MF Minelli C Zhao Q Lawlor DA Sheehan NA. Improving the accuracy of two-sample summary-data Mendelian randomization: moving beyond the NOME assumption. Int J Epidemiol. (2019) 48:728–42. doi: 10.1093/ije/dyy258 PMC6659376 30561657

[23] Lee Y. Role of vitamin C in targeting cancer stem cells and cellular plasticity. Cancers (Basel). (2023) 15:5657. doi: 10.3390/cancers15235657 38067361 PMC10705783

[24] El Deib MM El-Sharkawy NI Beheiry RR. Boldenone undecylenate disrupts the immune system and induces autoimmune clinical hypothyroidism in rats: Vitamin C ameliorative effects. Int Immunopharmacol. (2021) 99:107939. doi: 10.1016/j.intimp.2021.107939 34224995

[25] Karimi F Omrani GR. Effects of selenium and vitamin C on the serum level of antithyroid peroxidase antibody in patients with autoimmune thyroiditis. J Endocrinol Invest. (2019) 42:481–7. doi: 10.1007/s40618-018-0944-7 30182359

[26] Chainy GBN Sahoo DK. Hormones and oxidative stress: an overview. Free Radic Res. (2020) 54:1–26. doi: 10.1080/10715762.2019.1702656 31868060

[27] Wang J Wan K Chang X. Association of autoimmune thyroid disease with type 1 diabetes mellitus and its ultrasonic diagnosis and management. World J Diabetes. (2024) 15:348–60. doi: 10.4239/wjd.v15.i3.348 PMC10999045 38591076

[28] Tronci L Serreli G Piras C. Vitamin C cytotoxicity and its effects in redox homeostasis and energetic metabolism in papillary thyroid carcinoma cell lines. Antioxid (Basel). (2021) 10:809. doi: 10.3390/antiox10050809 PMC8161084 34065197

[29] Carazo A Macáková K Matoušová K. Vitamin A update: forms, sources, kinetics, detection, function, deficiency, therapeutic use and toxicity. Nutrients. (2021) 13:1703. doi: 10.3390/nu13051703 34069881 PMC8157347

[30] Haugen BR. The effect of vitamin a, retinoids and retinoid receptors on the hypothalamic-pituitary-thyroid axis. In: Beck-Peccoz P, editor. Syndromes of hormone resistance on the hypothalamic-Pituitary-Thyroid axis. Endocrine Updates. Springer, Boston, MA (2004). p. 149–63.

[31] Farasati Far B Broomand Lomer N Gharedaghi H. Is beta-carotene consumption associated with thyroid hormone levels? Front Endocrinol (Lausanne). (2023) 14:1089315. doi: 10.3389/fendo.2023.1089315 37305054 PMC10250628

[32] Hossein-nezhad A Spira A Holick MF. Influence of vitamin D status and vitamin D3 supplementation on genome wide expression of white blood cells: a randomized double-blind clinical trial. PloS One. (2013) 8:e58725. doi: 10.1371/journal.pone.0058725 23527013 PMC3604145

[33] Carlberg C. Vitamin D: A micronutrient regulating genes. Curr Pharm Des. (2019) 25:1740–6. doi: 10.2174/1381612825666190705193227 31298160

[34] Stöcklin E Eggersdorfer M. Vitamin D, an essential nutrient with versatile functions in nearly all organs. Int J Vitam Nutr Res. (2013) 83:92–100. doi: 10.1024/0300-9831/a000151 24491882

[35] Lebiedziński F Lisowska KA. Impact of vitamin D on immunopathology of hashimoto’s thyroiditis: from theory to practice. Nutrients. (2023) 15:3174. doi: 10.3390/nu15143174 37513592 PMC10385100

[36] Misharin A Hewison M Chen CR. Vitamin D deficiency modulates Graves’ hyperthyroidism induced in BALB/c mice by thyrotropin receptor immunization. Endocrinology. (2009) 150:1051–60. doi: 10.1210/en.2008-1191 PMC2646531 18927213

[37] Waterhouse M Pham H Rahman ST. The effect of vitamin D supplementation on hypothyroidism in the randomized controlled D-health trial. Thyroid. (2023) 33:1302–10. doi: 10.1089/thy.2023.0317 37698908

[38] Grove-Laugesen D Ebbehoj E Watt T. Effect of vitamin D supplementation on graves’ Disease: the DAGMAR trial. Thyroid. (2023) 33:1110–8. doi: 10.1089/thy.2023.0111 37218433

[39] Zhao J Wang H Zhang Z. Vitamin D deficiency as a risk factor for thyroid cancer: A meta-analysis of case-control studies. Nutrition. (2019) 57:5–11. doi: 10.1016/j.nut.2018.04.015 30086436

[40] Peng W Wang K Zheng R. 1,25 dihydroxyvitamin D3 inhibits the proliferation of thyroid cancer stem-like cells via cell cycle arrest. Endocr Res. (2016) 41:71–80. doi: 10.3109/07435800.2015.1037048 27030645

[41] Chiang KC Kuo SF Chen CH. MART-10, the vitamin D analog, is a potent drug to inhibit anaplastic thyroid cancer cell metastatic potential. Cancer Lett. (2015) 369:76–85. doi: 10.1016/j.canlet.2015.07.024 26282787

[42] Chandler PD Chen WY Ajala ON. Effect of Vitamin D3 Supplements on Development of Advanced Cancer: A Secondary Analysis of the VITAL Randomized Clinical Trial [published correction appears in JAMA Netw Open. JAMA Netw Open. (2020) 3:e2025850. doi: 10.1001/jamanetworkopen.2020.25850 33206192 PMC7675103

[43] Choi YM Kim WG Kim TY. Serum vitamin D3 levels are not associated with thyroid cancer prevalence in euthyroid subjects without autoimmune thyroid disease. Korean J Intern Med. (2017) 32:102–8. doi: 10.3904/kjim.2015.090 PMC5214716 27581957

[44] Fan J Fu S Chen X. Thyroid nodules and its association with vitamin D in centenarians. Exp Gerontol. (2022) 161:111730. doi: 10.1016/j.exger.2022.111730 35134474

[45] Bolat H Erdoğan A. Benign nodules of the thyroid gland and 25-hydroxy-vitamin D levels in euthyroid patients. Ann Saudi Med. (2022) 42:83–8. doi: 10.5144/0256-4947.2022.83 PMC8982002 35380060

[46] Venditti P Di Stefano L Di Meo S. Vitamin E management of oxidative damage-linked dysfunctions of hyperthyroid tissues. Cell Mol Life Sci. (2013) 70:3125–44. doi: 10.1007/s00018-012-1217-9 PMC11114018 23255045

[47] Lima-Antoine L de Sousa Alves Neri JL de Melo TCT. Histopathological prognosis of papillary thyroid carcinoma associated with nutritional status of vitamins A and E. Eur J Clin Nutr. (2022) 76:469–76. doi: 10.1038/s41430-021-00976-5 34230633

[48] Tajima A Kubo Y Horiguchi S. et al relationship between serum homocysteine concentration and dietary factors in young Japanese women. Nutrients. (2023) 15:4740. doi: 10.3390/nu15224740 38004134 PMC10675237

[49] Najafipour R Moghbelinejad S Aleyasin A Jalilvand A. Effect of B9 and B12 vitamin intake on semen parameters and fertility of men with MTHFR polymorphisms. Andrology. (2017) 5:704–10. doi: 10.1111/andr.12351 28440964

[50] Ding X Wang Y Liu J. Impaired sensitivity to thyroid hormones is associated with elevated homocysteine levels in the euthyroid population. J Clin Endocrinol Metab. (2022) 107:e3731–7. doi: 10.1210/clinem/dgac371 35708733

