# Supplementary material for: Association of circulating vitamin levels with thyroid diseases: a Mendelian randomization study
Source: Front Endocrinol (Lausanne). 2024 Jun 11;15:1360851. doi: 10.3389/fendo.2024.1360851 (PMC11196410; doi:10.3389/fendo.2024.1360851)
Supplement: Supplementary file 3 [file DataSheet_3.docx]

***Supplementary Figure S1-S30:***

| a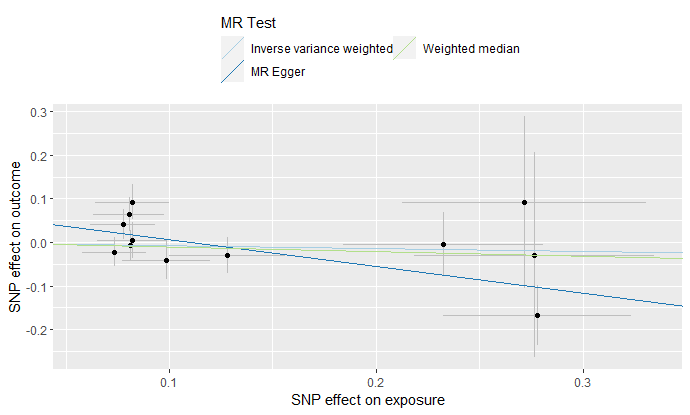 | b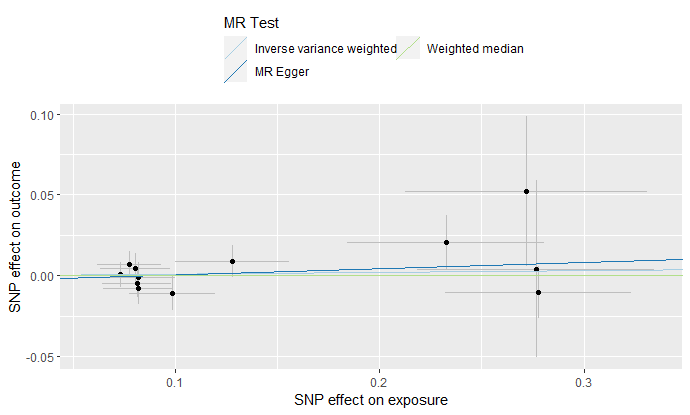 |
| --- | --- |
| c  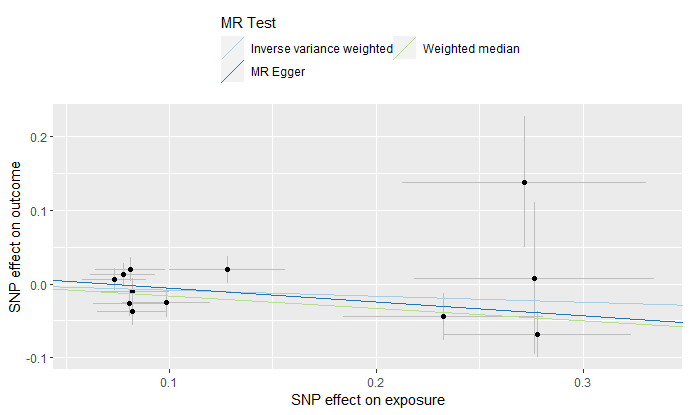 | d  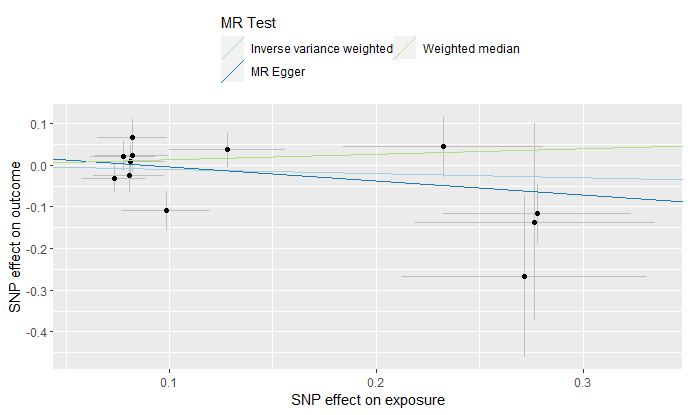 |

**Supplementary Figure. S1** The estimation of pleiotropy for exposure(Circulating vitamin A level)and outcome(thyroid diseases) using MR-Egger intercept. (a) Autoimmune hyperthyroidism. (b) Autoimmune hypothyroidism. (c) Nontoxic goitre/thyroid nodule. (d)Malignant neoplasm of thyroid gland.

| a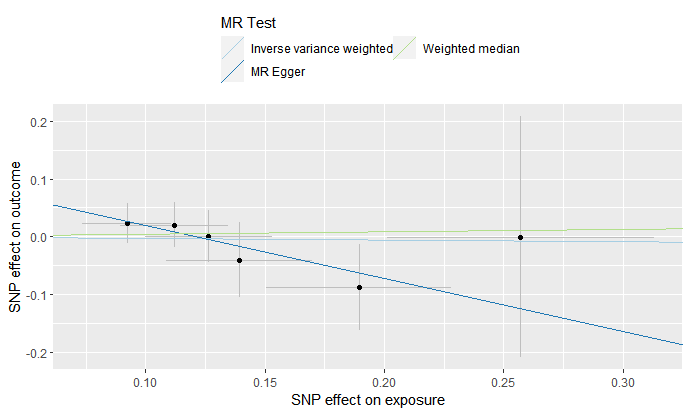 | b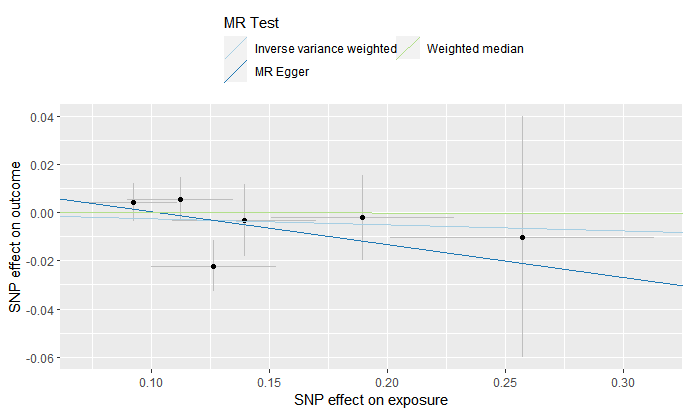 |
| --- | --- |
| c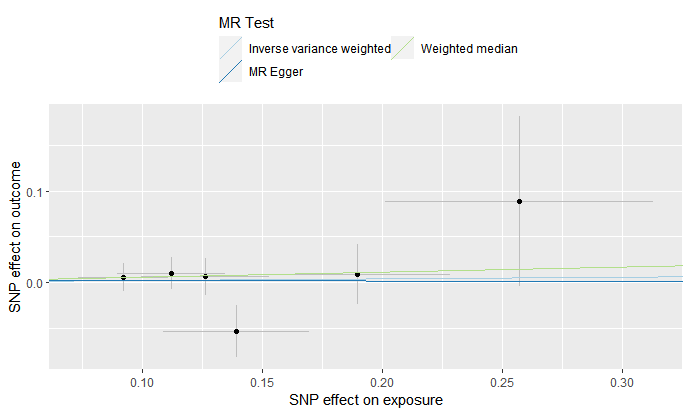 | d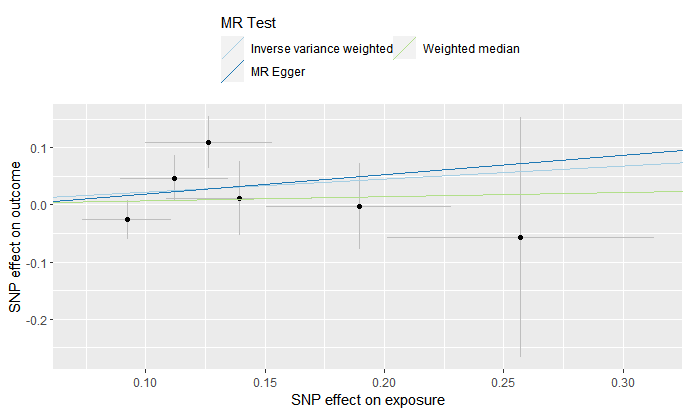 |

**Supplementary Figure. S2** The estimation of pleiotropy for exposure(Circulating vitamin B9 level)and outcome(thyroid diseases) using MR-Egger intercept. (a) Autoimmune hyperthyroidism. (b) Autoimmune hypothyroidism. (c) Nontoxic goitre/thyroid nodule. (d)Malignant neoplasm of thyroid gland.

| a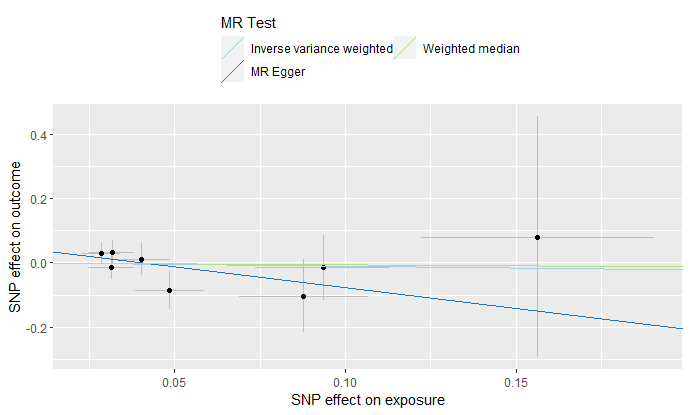 | b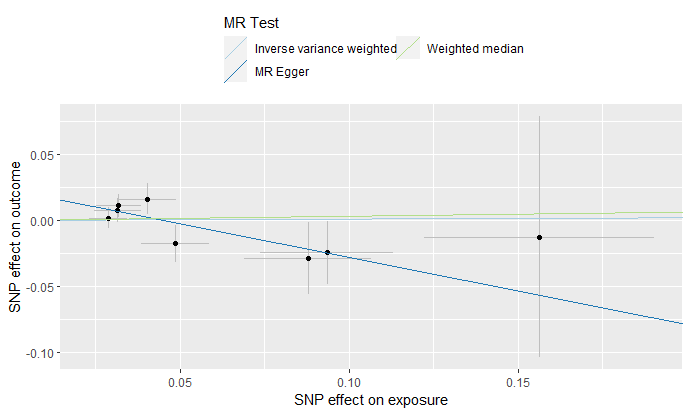 |
| --- | --- |
| c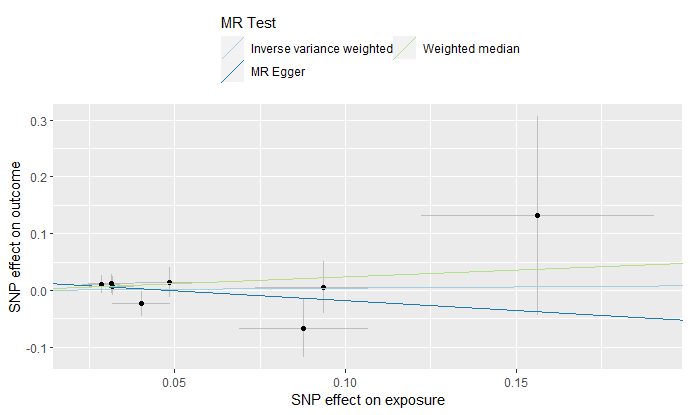 | d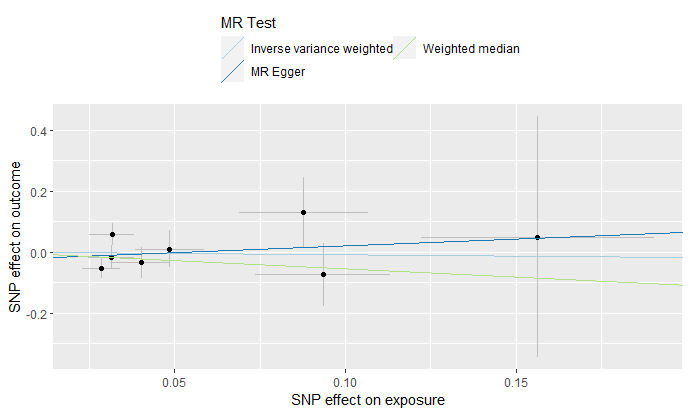 |

**Supplementary Figure. S3** The estimation of pleiotropy for exposure(Circulating vitamin B12 level)and outcome(thyroid diseases) using MR-Egger intercept. (a) Autoimmune hyperthyroidism. (b) Autoimmune hypothyroidism. (c) Nontoxic goitre/thyroid nodule. (d)Malignant neoplasm of thyroid gland.

| a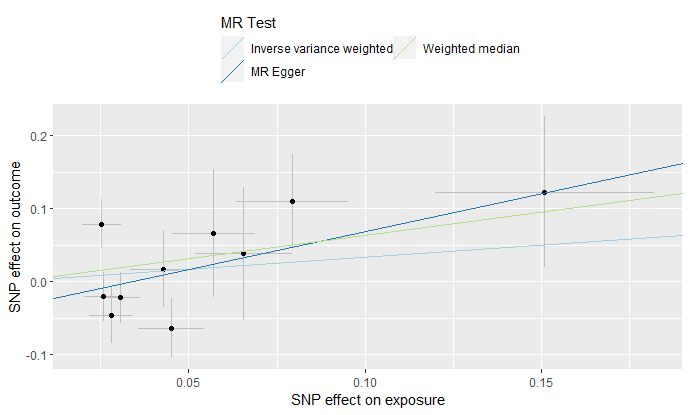 | b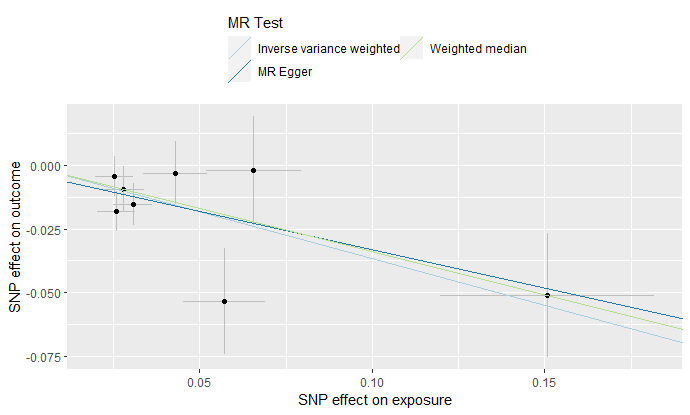 |
| --- | --- |
| c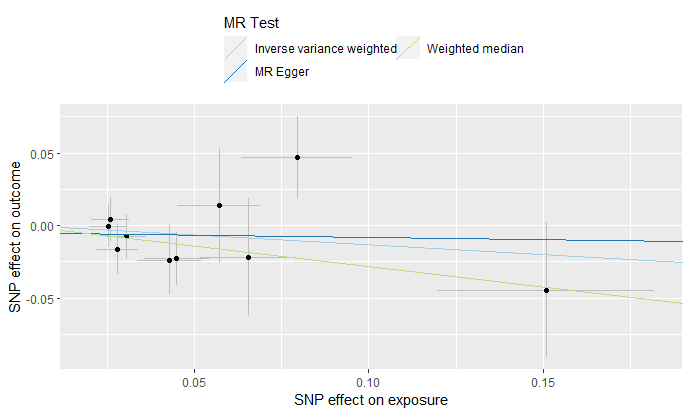 | d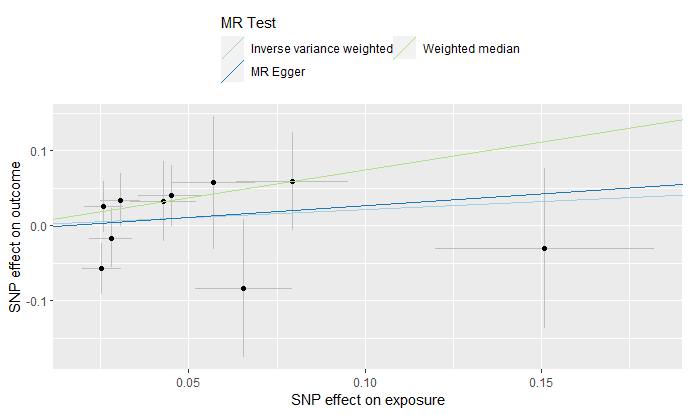 |

**Supplementary Figure. S4** The estimation of pleiotropy for exposure(Circulating vitamin C level)and outcome(thyroid diseases) using MR-Egger intercept. (a) Autoimmune hyperthyroidism. (b) Autoimmune hypothyroidism. (c) Nontoxic goitre/thyroid nodule. (d)Malignant neoplasm of thyroid gland.

| a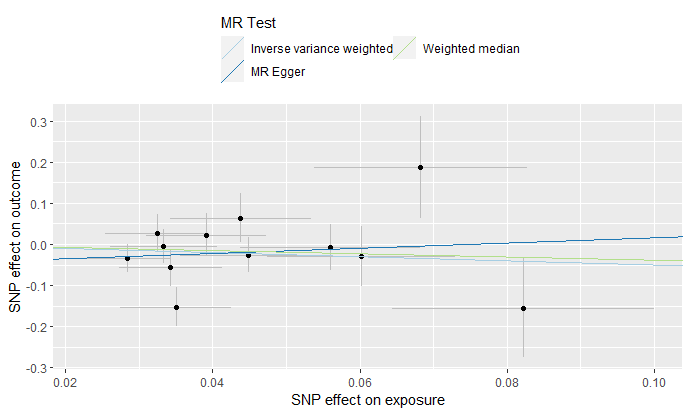 | b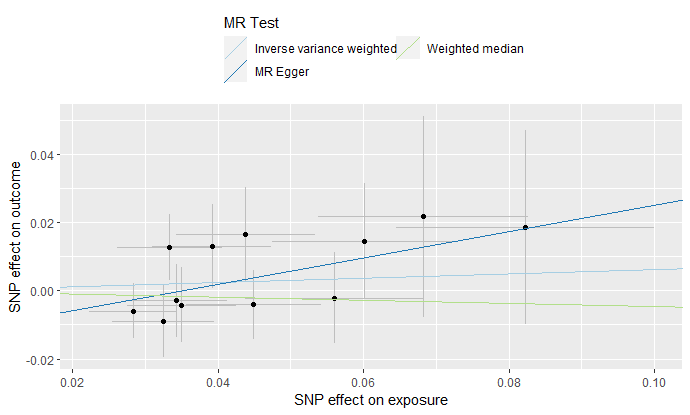 |
| --- | --- |
| c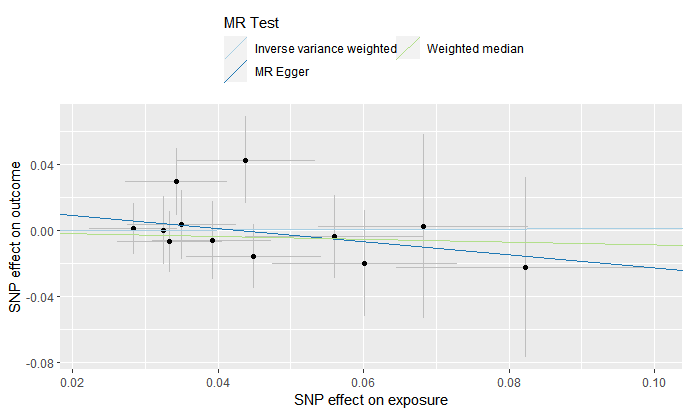 | d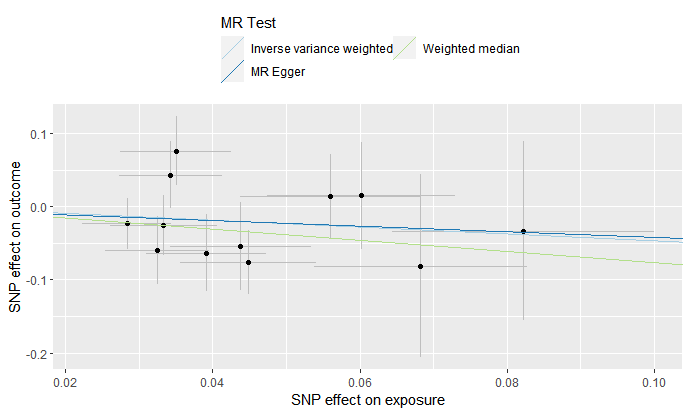 |

**Supplementary Figure. S5** The estimation of pleiotropy for exposure(Circulating vitamin D level)and outcome(thyroid diseases) using MR-Egger intercept. (a) Autoimmune hyperthyroidism. (b) Autoimmune hypothyroidism. (c) Nontoxic goitre/thyroid nodule. (d)Malignant neoplasm of thyroid gland.

| a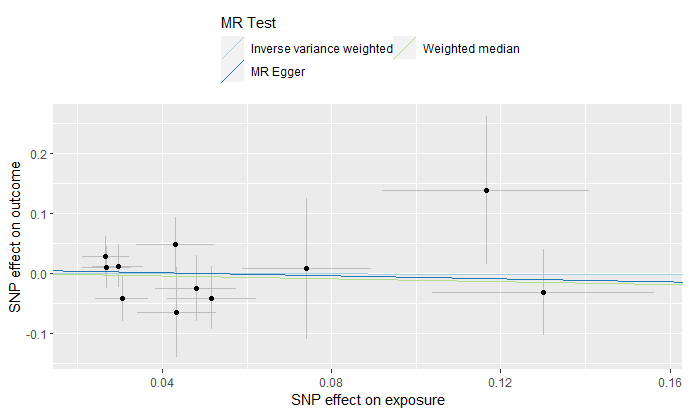 | b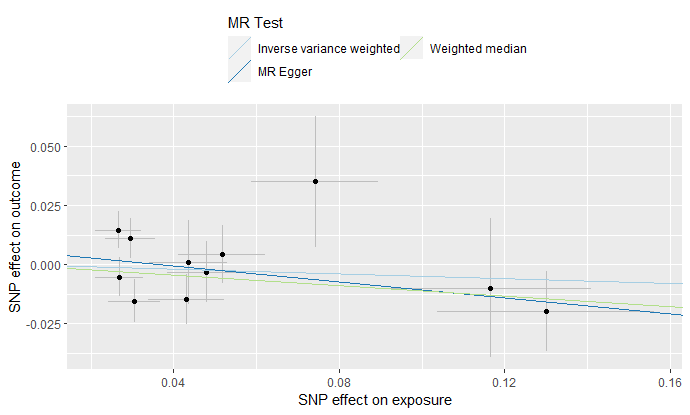 |
| --- | --- |
| c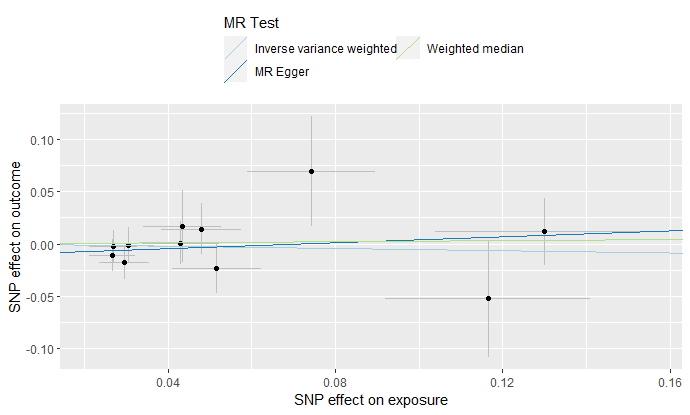 | d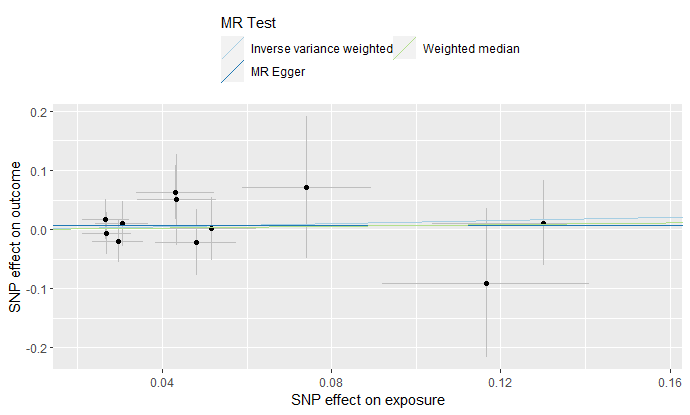 |

**Supplementary Figure. S6** The estimation of pleiotropy for exposure(Circulating vitamin E level)and outcome(thyroid diseases) using MR-Egger intercept. (a) Autoimmune hyperthyroidism. (b) Autoimmune hypothyroidism. (c) Nontoxic goitre/thyroid nodule. (d)Malignant neoplasm of thyroid gland.

| a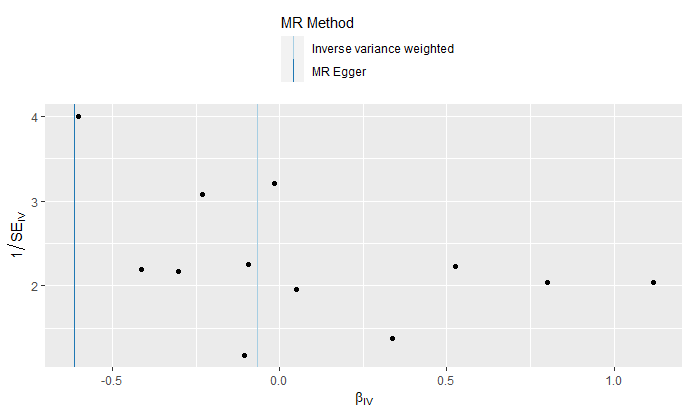 | b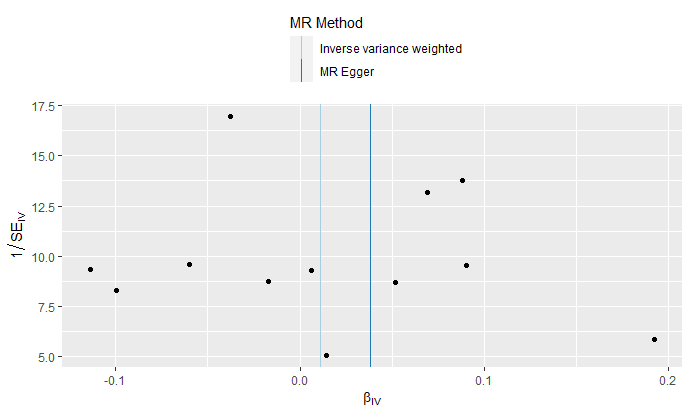 |
| --- | --- |
| c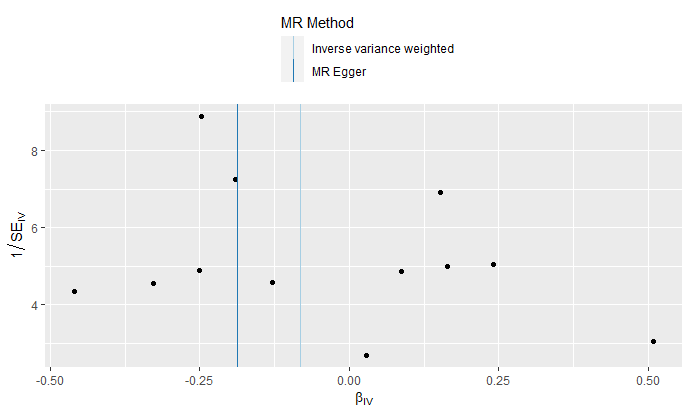 | d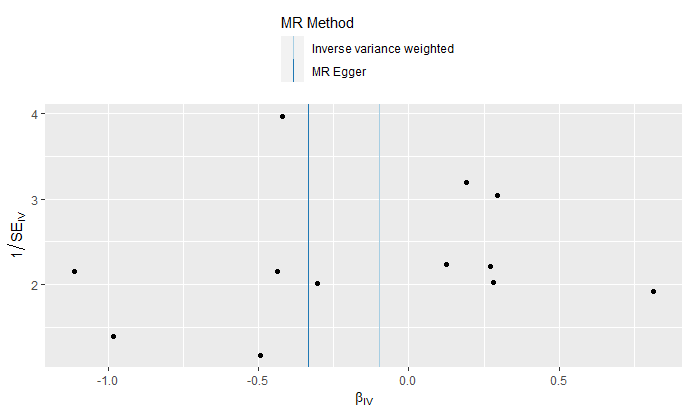 |

**Supplementary Figure. S7** The estimation of heterogeneity for exposure(Circulating vitamin A level)and outcome(thyroid diseases) using Funnel plot.(a) Autoimmune hyperthyroidism. (b) Autoimmune hypothyroidism. (c) Nontoxic goitre/thyroid nodule. (d)Malignant neoplasm of thyroid gland.

| a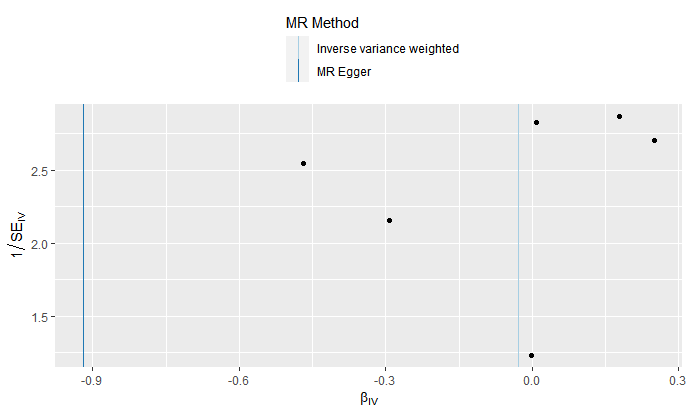 | b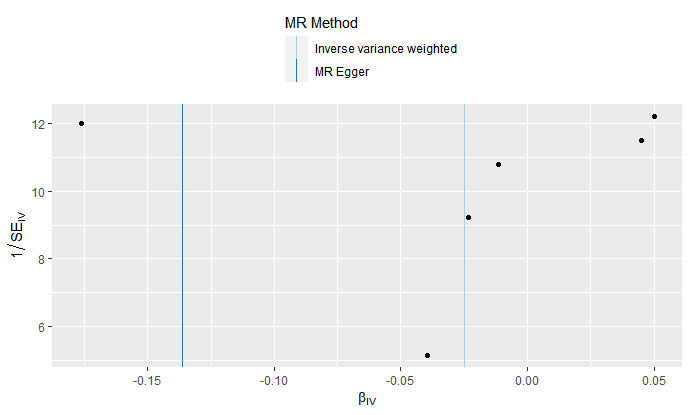 |
| --- | --- |
| c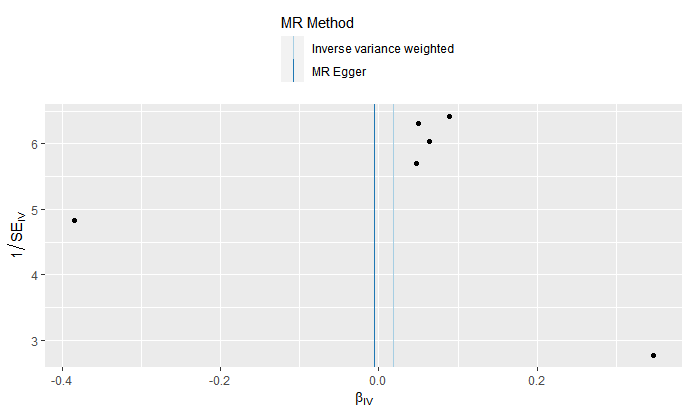 | d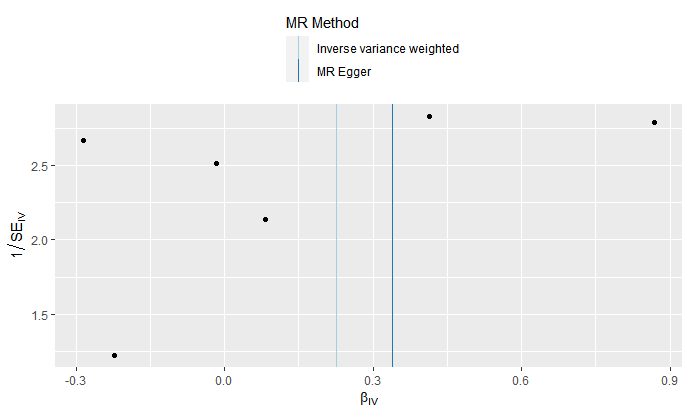 |

**Supplementary Figure. S8** The estimation of heterogeneity for exposure(Circulating vitamin B9 level)and outcome(thyroid diseases) using Funnel plot.(a) Autoimmune hyperthyroidism. (b) Autoimmune hypothyroidism. (c) Nontoxic goitre/thyroid nodule. (d)Malignant neoplasm of thyroid gland.

| a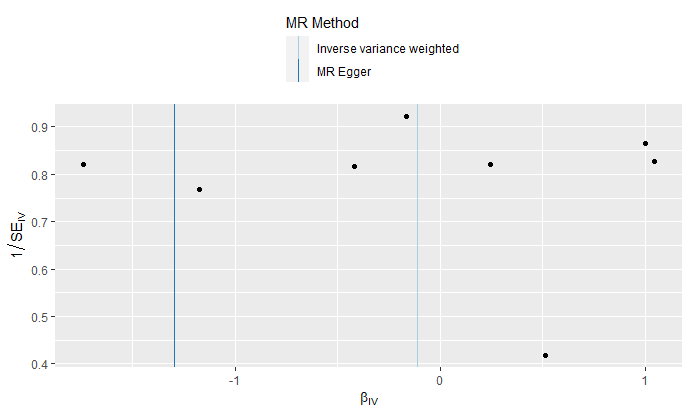 | b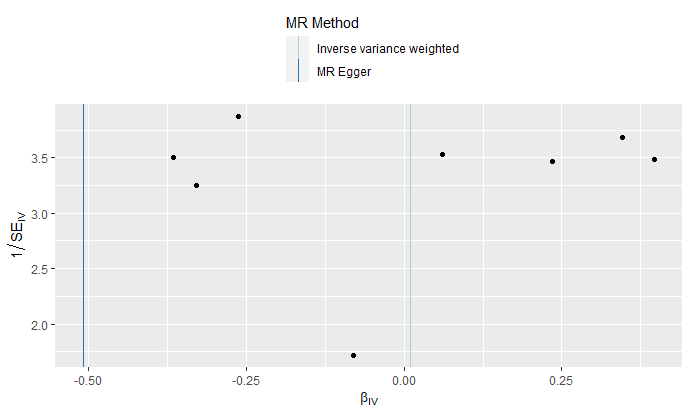 |
| --- | --- |
| c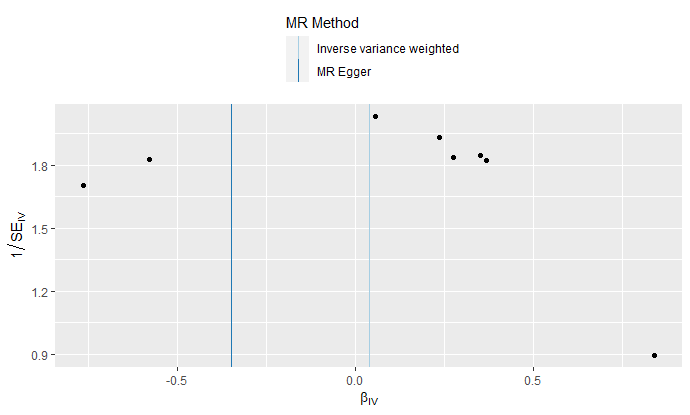 | d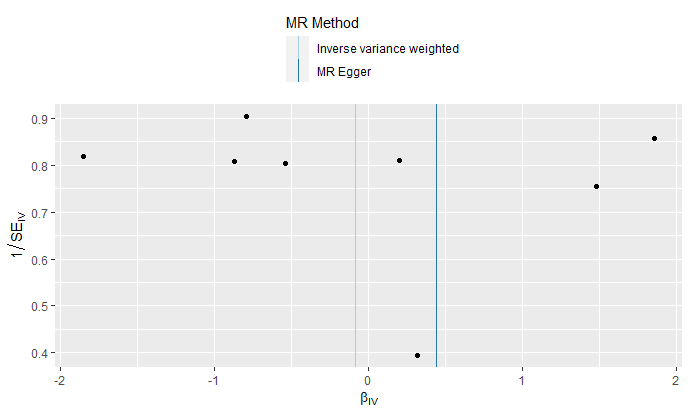 |

**Supplementary Figure. S9** The estimation of heterogeneity for exposure(Circulating vitamin B12 level)and outcome(thyroid diseases) using Funnel plot.(a) Autoimmune hyperthyroidism. (b) Autoimmune hypothyroidism. (c) Nontoxic goitre/thyroid nodule. (d)Malignant neoplasm of thyroid gland.

| a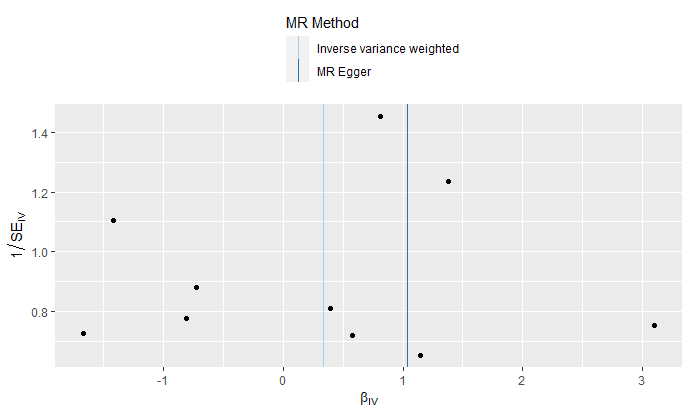 | b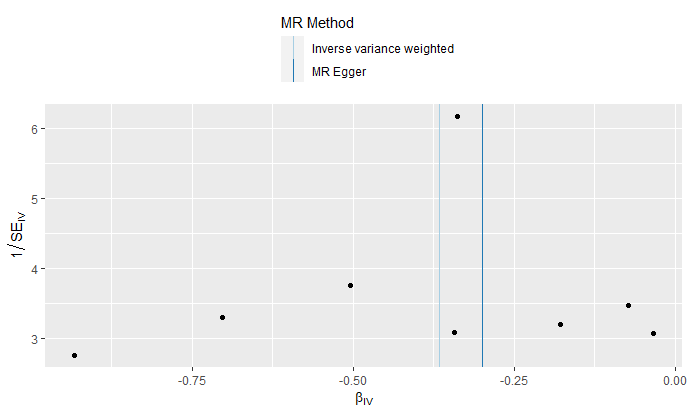 |
| --- | --- |
| c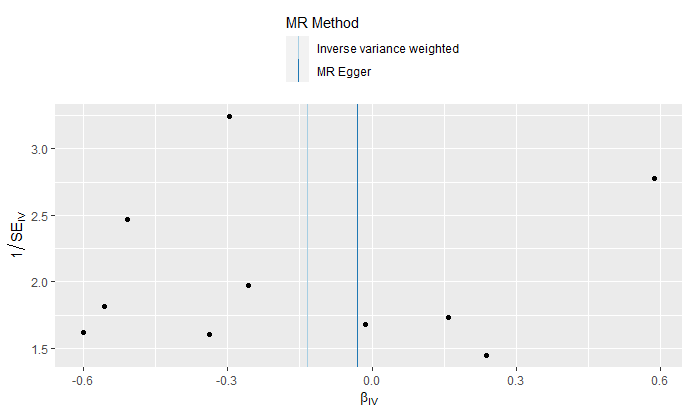 | d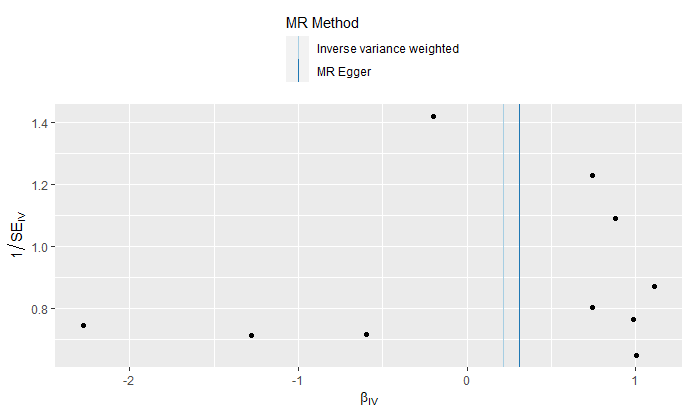 |

**Supplementary Figure. S10** The estimation of heterogeneity for exposure(Circulating vitamin C level)and outcome(thyroid diseases) using Funnel plot.(a) Autoimmune hyperthyroidism. (b) Autoimmune hypothyroidism. (c) Nontoxic goitre/thyroid nodule. (d)Malignant neoplasm of thyroid gland.

| a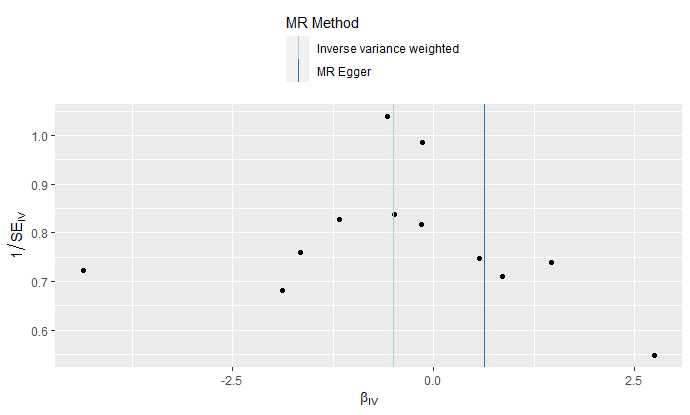 | b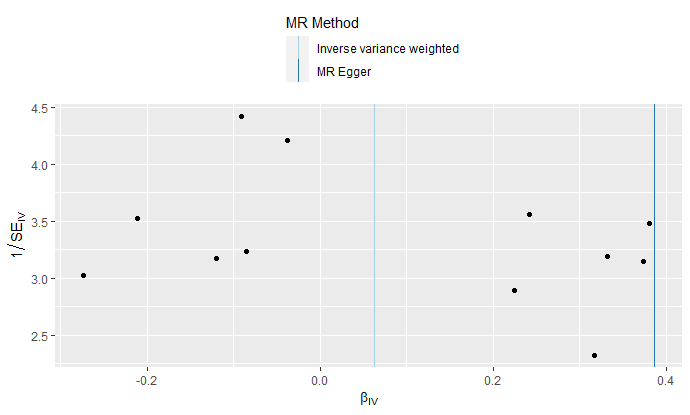 |
| --- | --- |
| c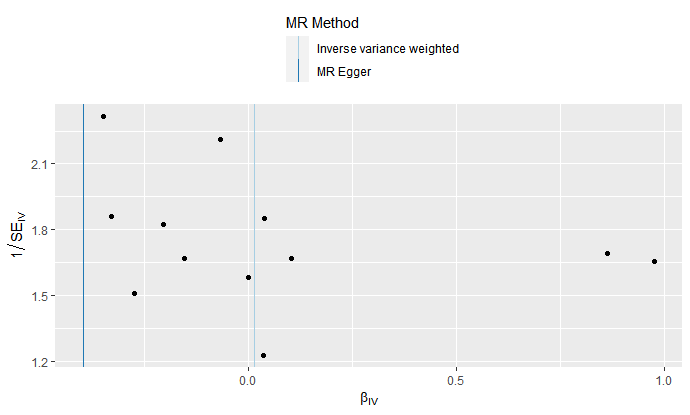 | d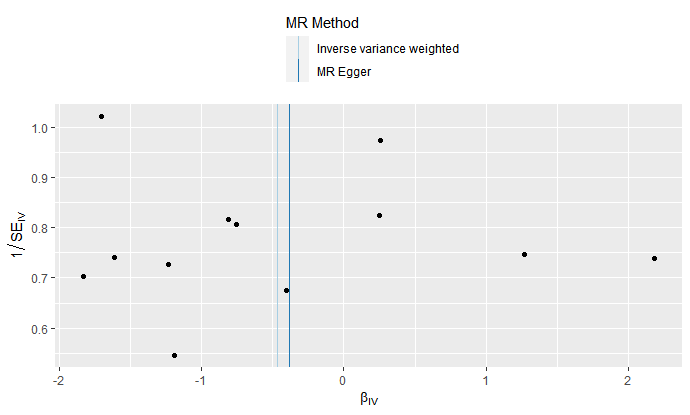 |

**Supplementary Figure. S11** The estimation of heterogeneity for exposure(Circulating vitamin D level)and outcome(thyroid diseases) using Funnel plot.(a) Autoimmune hyperthyroidism. (b) Autoimmune hypothyroidism. (c) Nontoxic goitre/thyroid nodule. (d)Malignant neoplasm of thyroid gland.

| a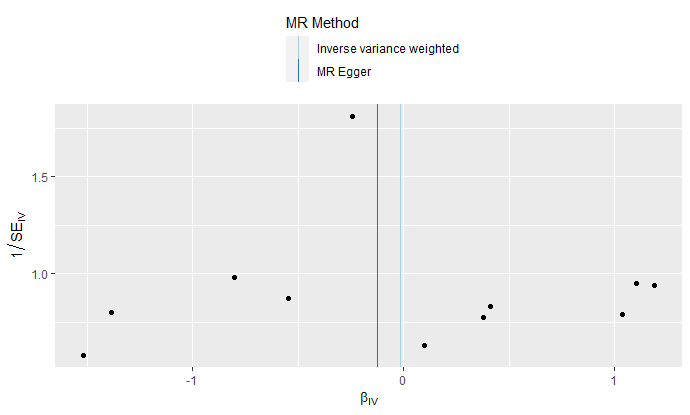 | b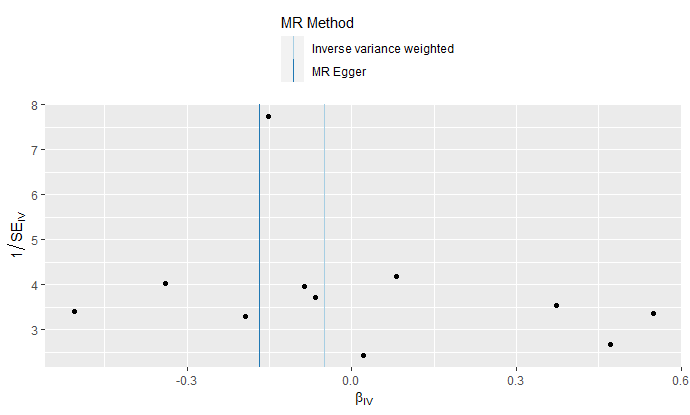 |
| --- | --- |
| c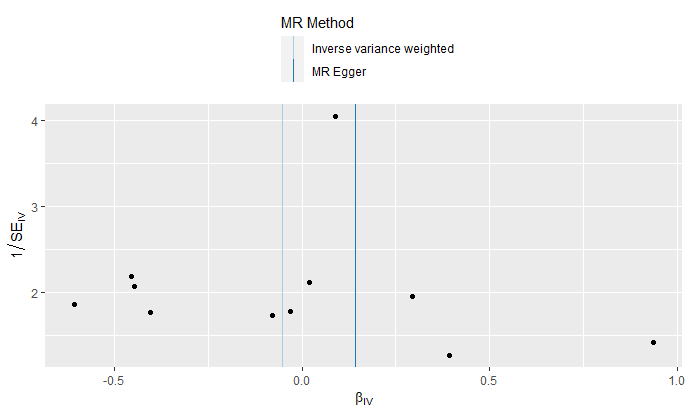 | d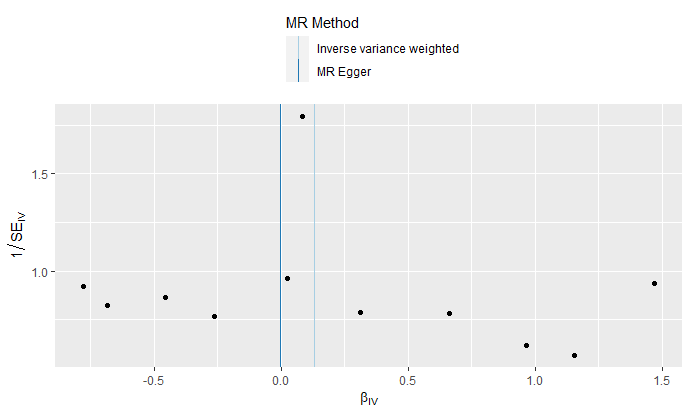 |

**Supplementary Figure. S12** The estimation of heterogeneity for exposure(Circulating vitamin E level)and outcome(thyroid diseases) using Funnel plot.(a) Autoimmune hyperthyroidism. (b) Autoimmune hypothyroidism. (c) Nontoxic goitre/thyroid nodule. (d)Malignant neoplasm of thyroid gland.

| a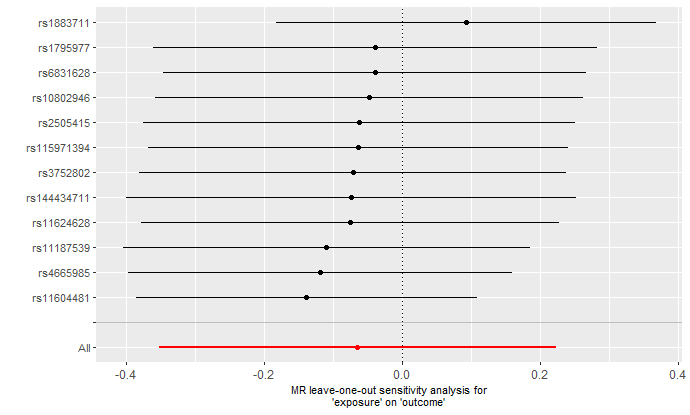 | b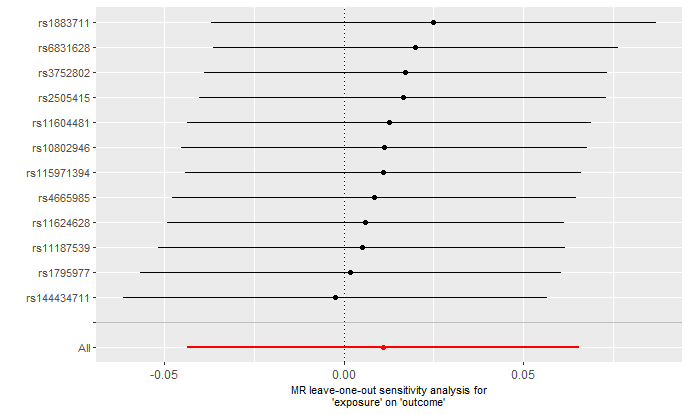 |
| --- | --- |
| c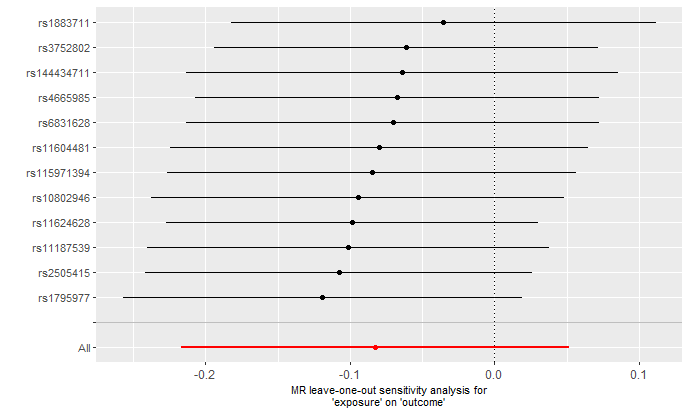 | d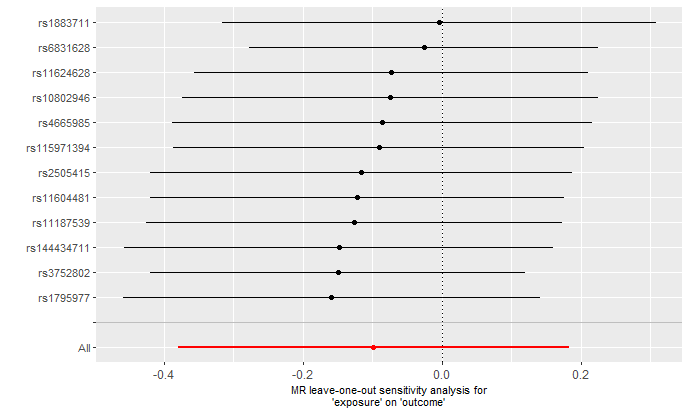 |

**Supplementary Figure. S13** MR leave-one-out sensitivity analysis for exposure(Circulating vitamin A level) on outcome(thyroid diseases). (a) Autoimmune hyperthyroidism. (b) Autoimmune hypothyroidism. (c) Nontoxic goitre/thyroid nodule. (d)Malignant neoplasm of thyroid gland.

| a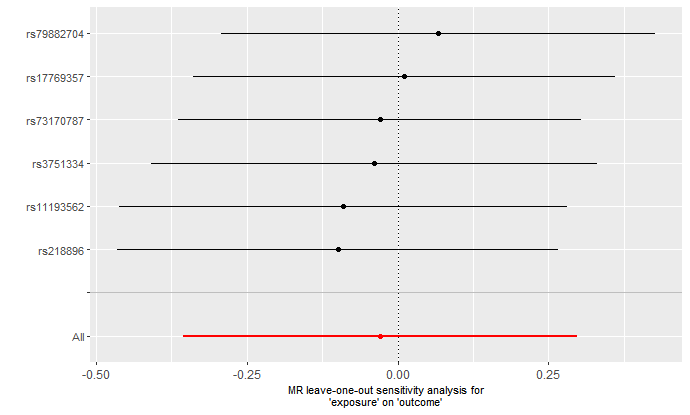 | b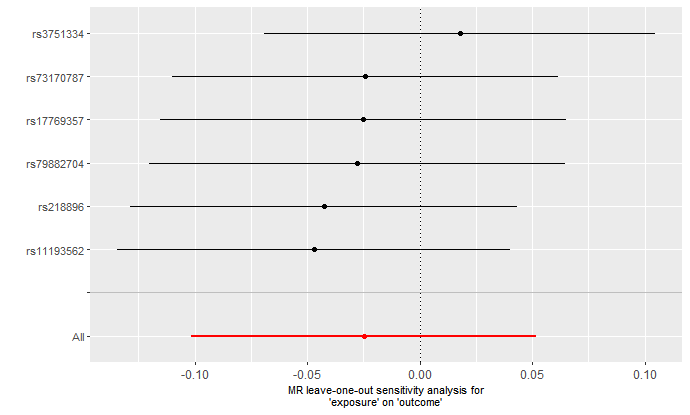 |
| --- | --- |
| c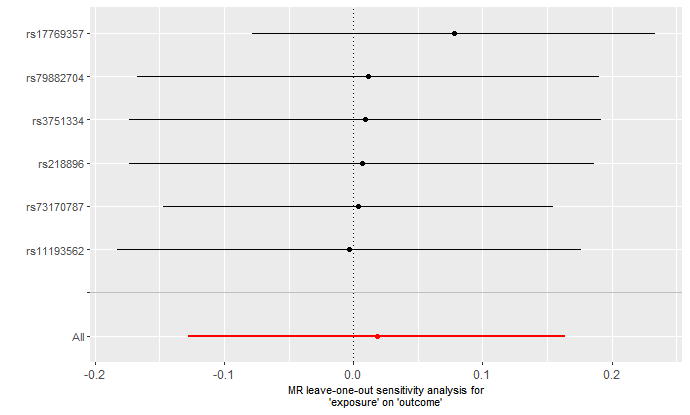 | d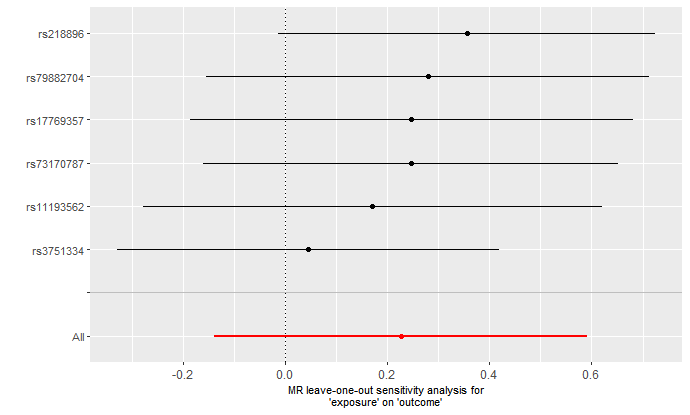 |

**Supplementary Figure. S14** MR leave-one-out sensitivity analysis for exposure(Circulating vitamin B9 level) on outcome(thyroid diseases). (a) Autoimmune hyperthyroidism. (b) Autoimmune hypothyroidism. (c) Nontoxic goitre/thyroid nodule. (d)Malignant neoplasm of thyroid gland.

| a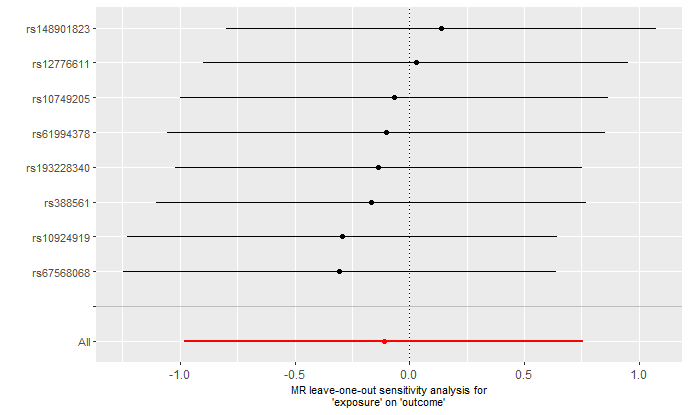 | b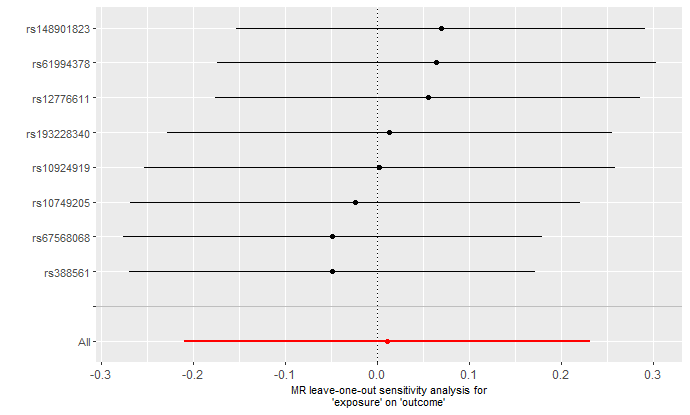 |
| --- | --- |
| c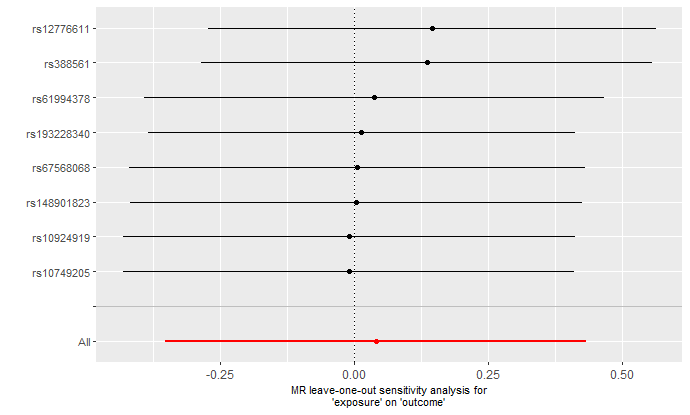 | d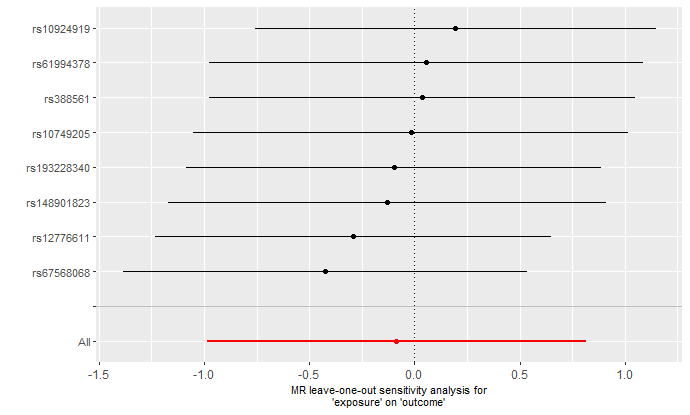 |

**Supplementary Figure. S15** MR leave-one-out sensitivity analysis for exposure(Circulating vitamin B12 levelv) on outcome(thyroid diseases). (a) Autoimmune hyperthyroidism. (b) Autoimmune hypothyroidism. (c) Nontoxic goitre/thyroid nodule. (d)Malignant neoplasm of thyroid gland.

| a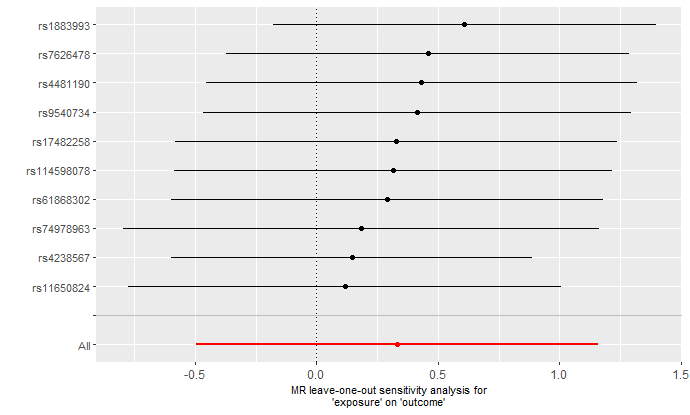 | b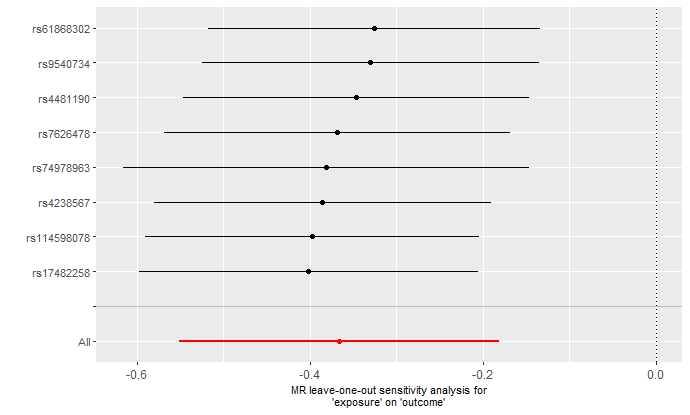 |
| --- | --- |
| c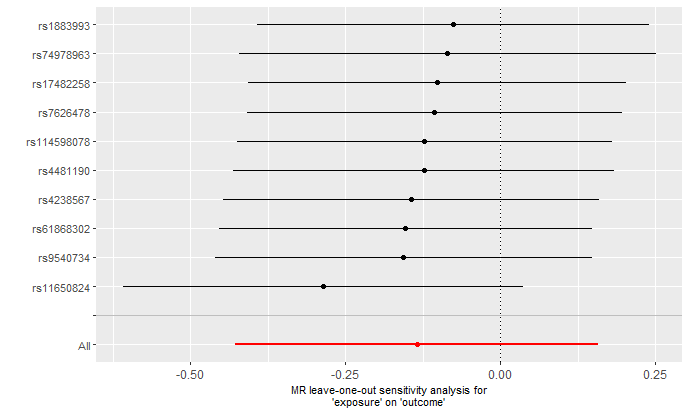 | d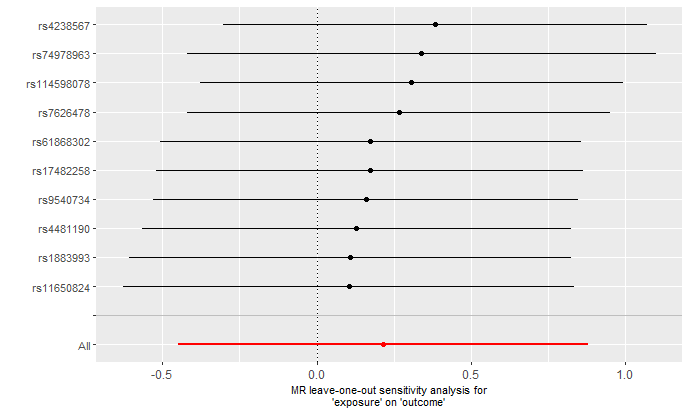 |

**Supplementary Figure. S16** MR leave-one-out sensitivity analysis for exposure(Circulating vitamin C level) on outcome(thyroid diseases). (a) Autoimmune hyperthyroidism. (b) Autoimmune hypothyroidism. (c) Nontoxic goitre/thyroid nodule. (d)Malignant neoplasm of thyroid gland.

| a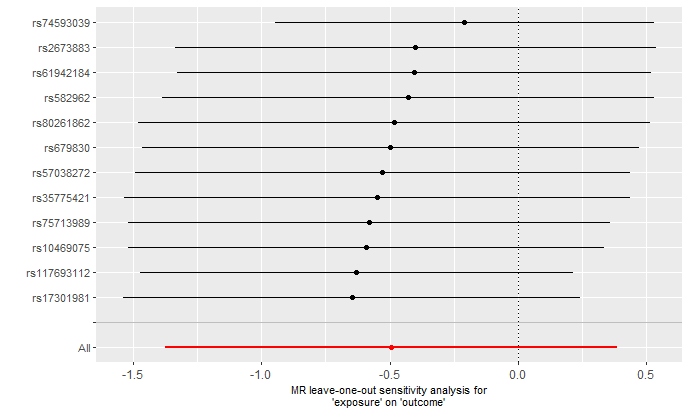 | b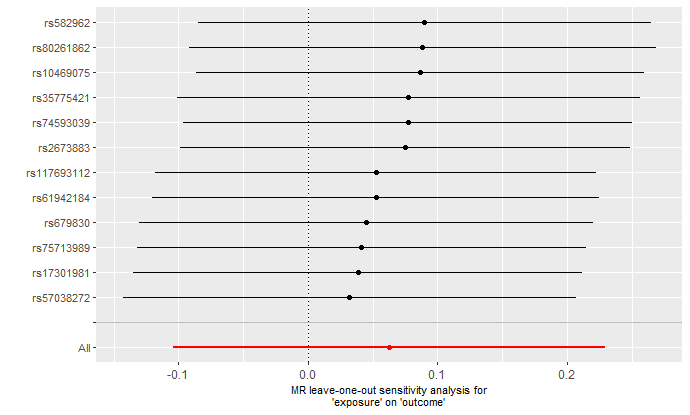 |
| --- | --- |
| c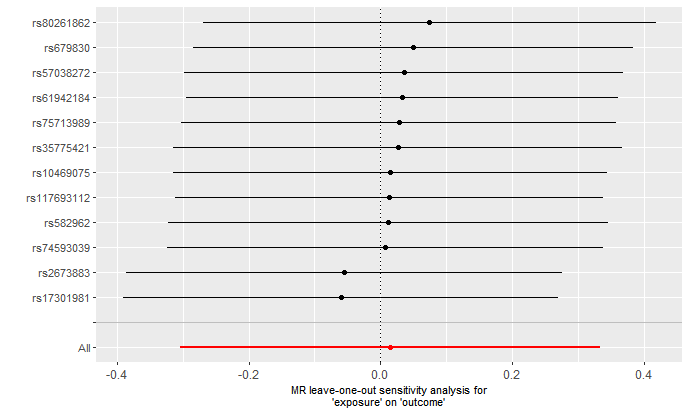 | d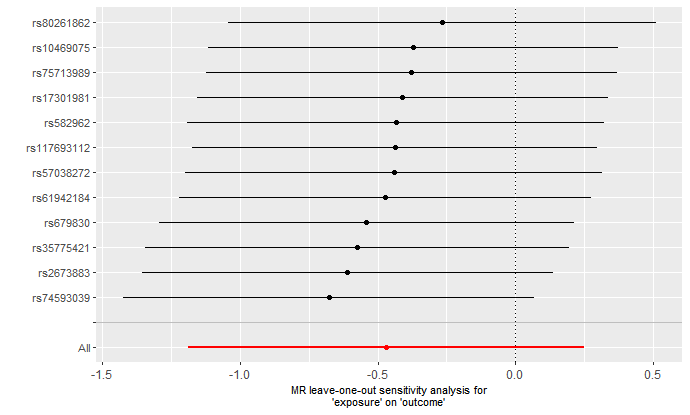 |

**Supplementary Figure. S17** MR leave-one-out sensitivity analysis for exposure(Circulating vitamin D level) on outcome(thyroid diseases). (a) Autoimmune hyperthyroidism. (b) Autoimmune hypothyroidism. (c) Nontoxic goitre/thyroid nodule. (d)Malignant neoplasm of thyroid gland.

| a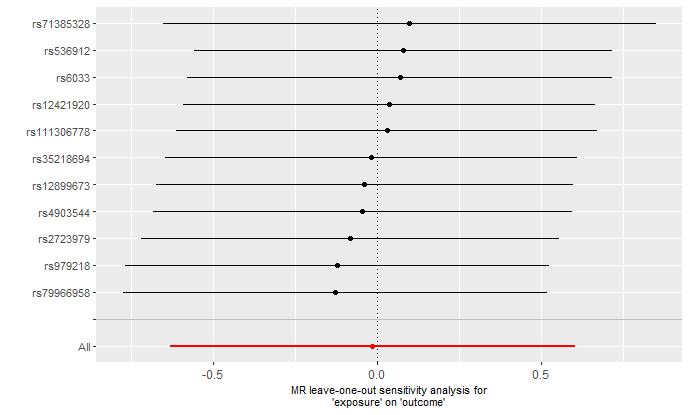 | b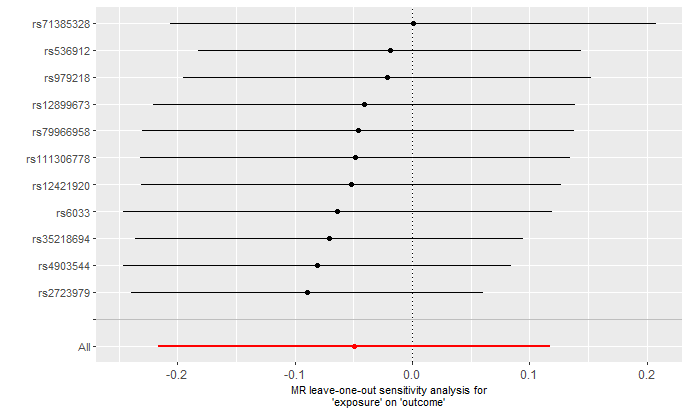 |
| --- | --- |
| c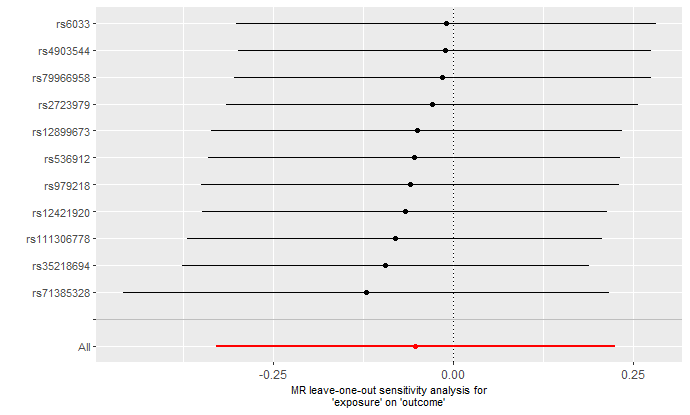 | d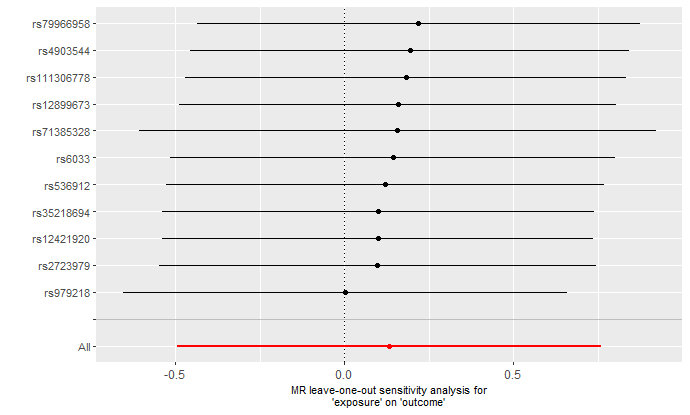 |

**Supplementary Figure. S18** MR leave-one-out sensitivity analysis for exposure(Circulating vitamin E level) on outcome(thyroid diseases). (a) Autoimmune hyperthyroidism. (b) Autoimmune hypothyroidism. (c) Nontoxic goitre/thyroid nodule. (d)Malignant neoplasm of thyroid gland.

| a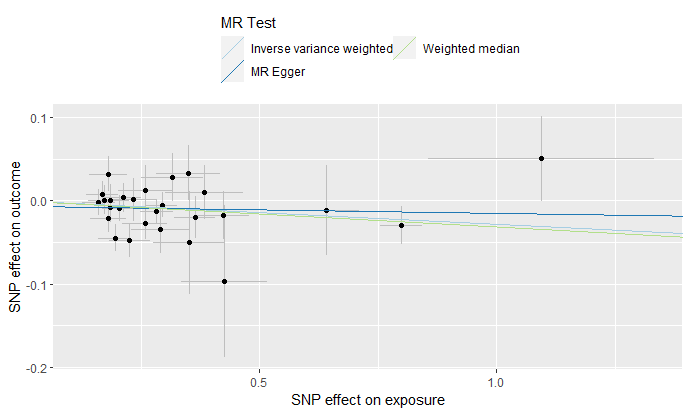 | b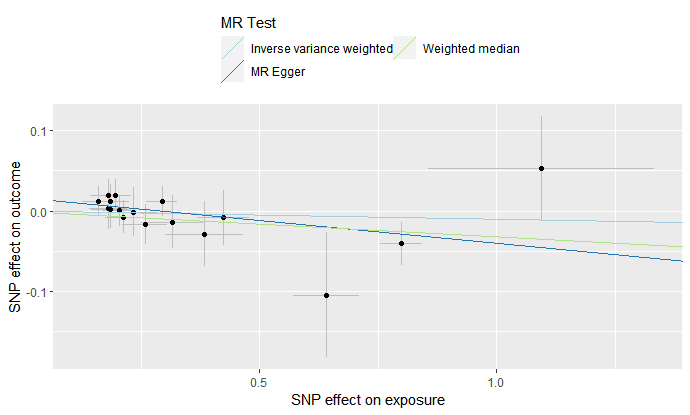 | c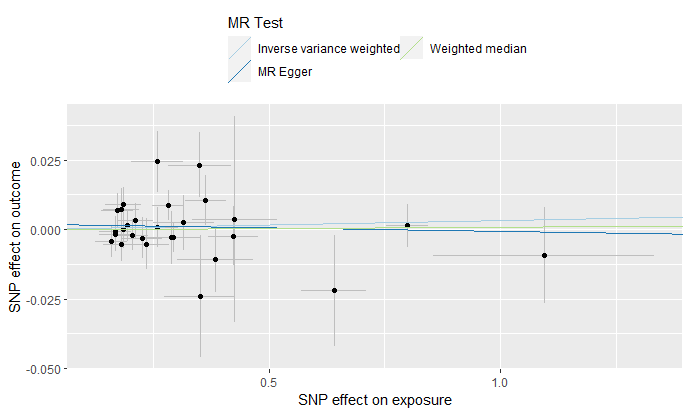 |
| --- | --- | --- |
| d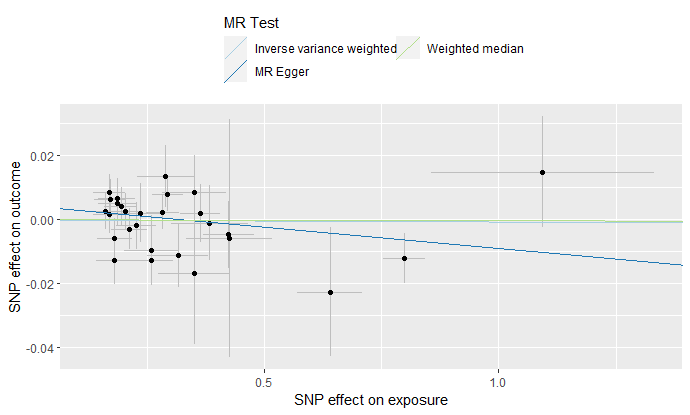 | e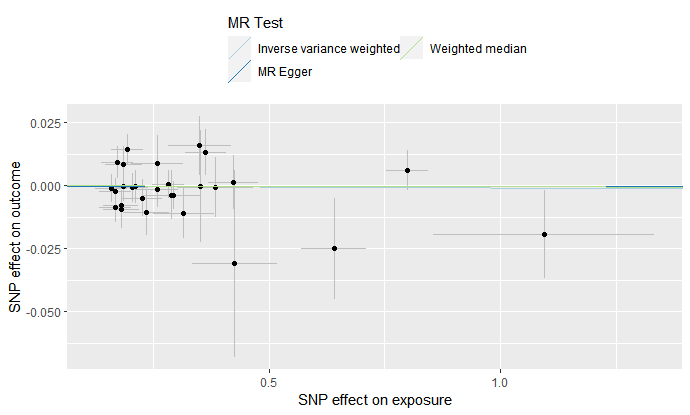 | f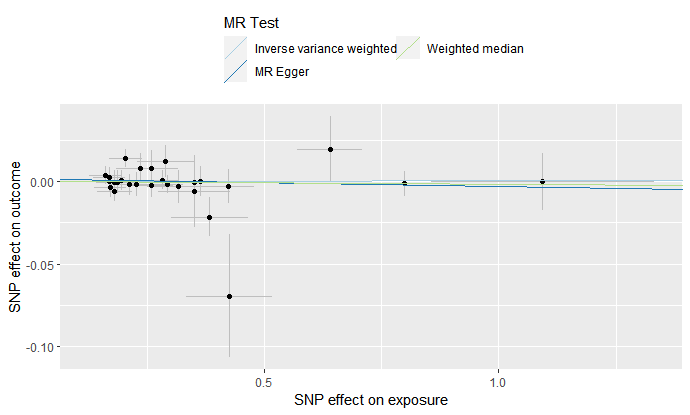 |

**Supplementary Figure. S19** The estimation of pleiotropy for exposure(Autoimmune hyperthyroidism) and outcome(Circulating vitamin levels) using MR-Egger intercept. (a) Vitamin A. (b)Vitamin B9. (c)Vitamin B12. (d) Vitamin C. (e)Vitamin D. (f) Vitamin E.

| a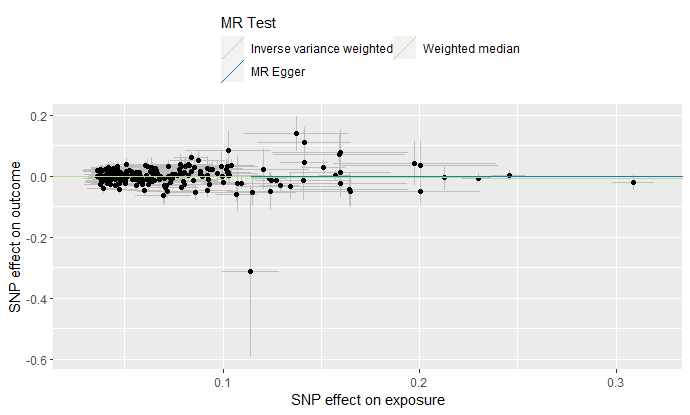 | b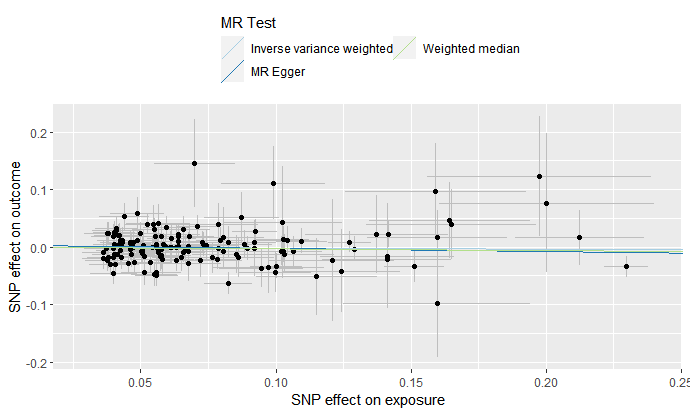 | c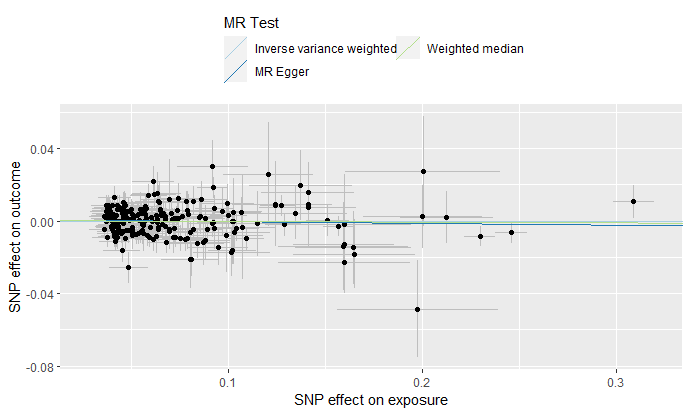 |
| --- | --- | --- |
| d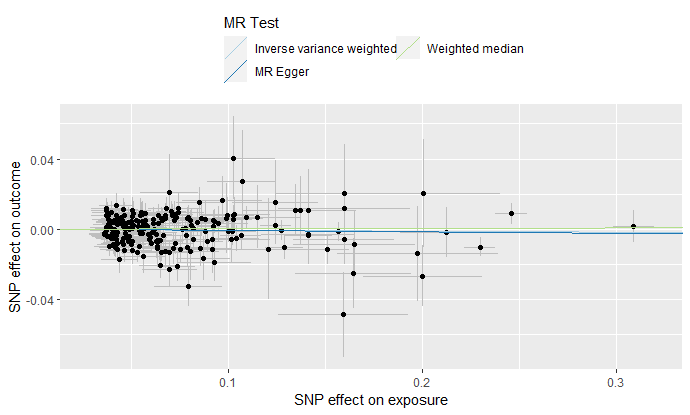 | e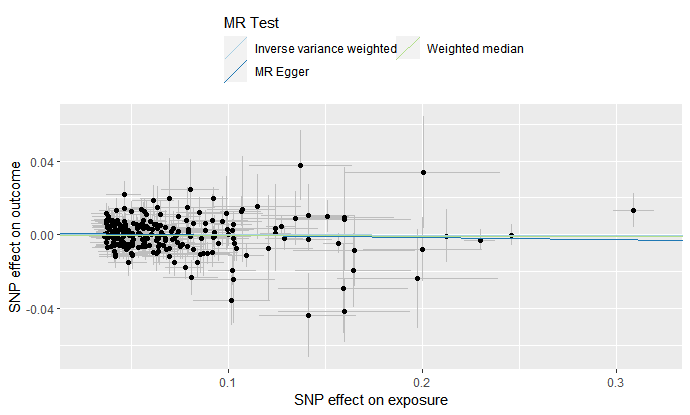 | d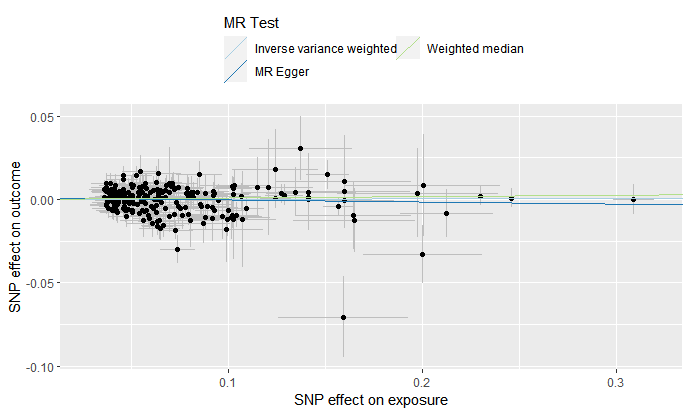 |

**Supplementary Figure. S20** The estimation of pleiotropy for exposure(Autoimmune hypothyroidism) and outcome(Circulating vitamin levels) using MR-Egger intercept. (a) Vitamin A. (b)Vitamin B9. (c)Vitamin B12. (d) Vitamin C. (e)Vitamin D. (f) Vitamin E.

| a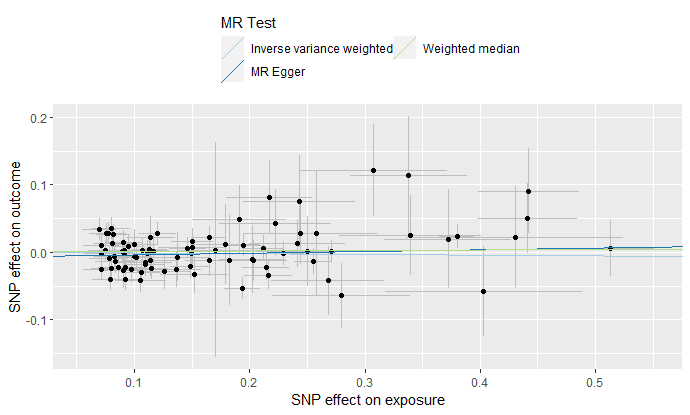 | b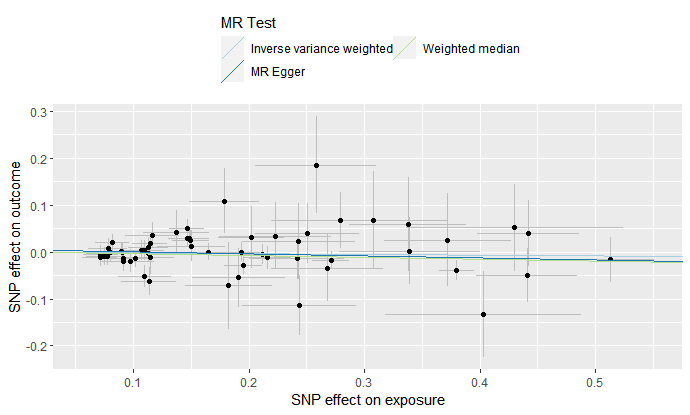 | c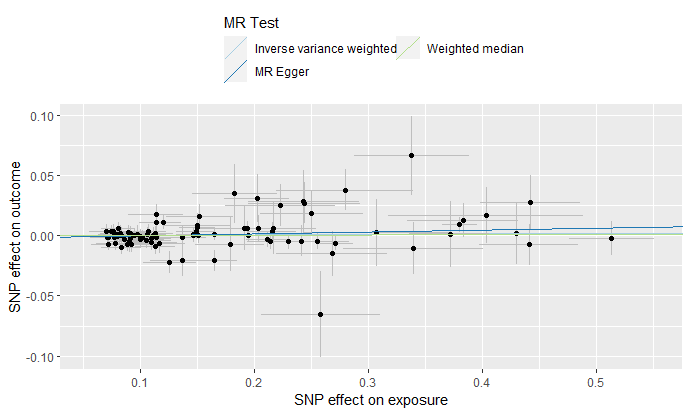 |
| --- | --- | --- |
| d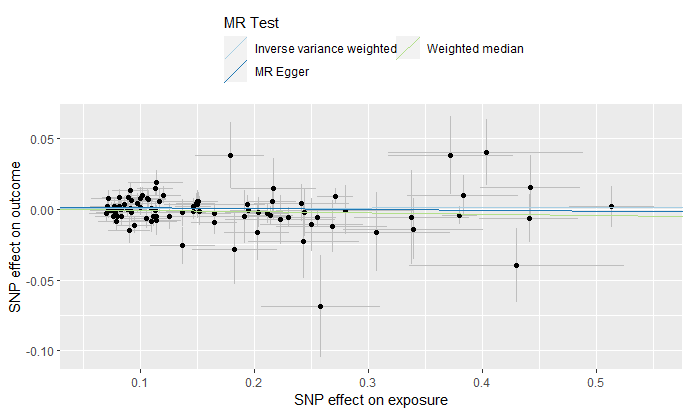 | e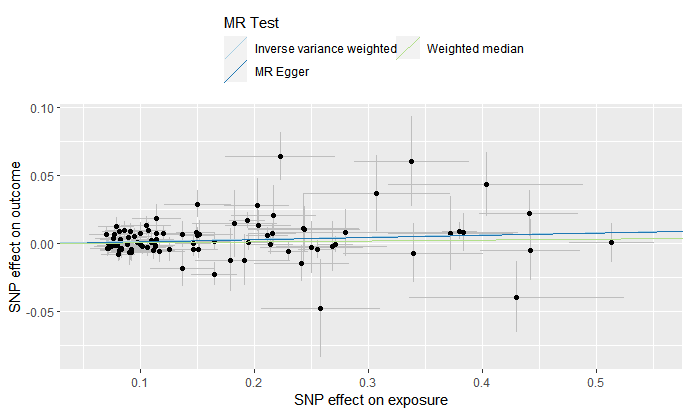 | f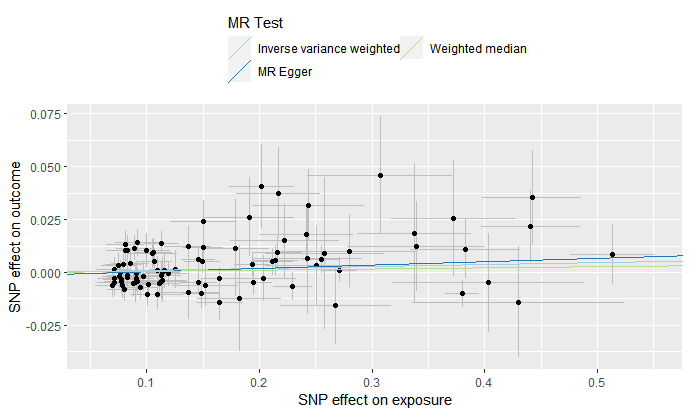 |

**Supplementary Figure. S21** The estimation of pleiotropy for exposure(Nontoxic goitre/Thyroid nodule) and outcome(Circulating vitamin levels) using MR-Egger intercept. (a) Vitamin A. (b)Vitamin B9. (c)Vitamin B12. (d) Vitamin C. (e)Vitamin D. (f) Vitamin E.

| a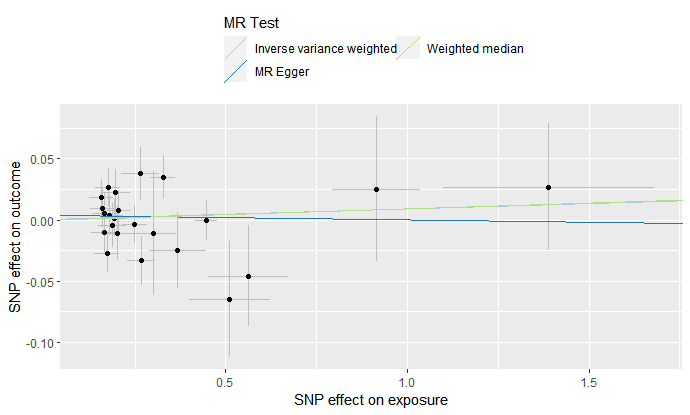 | b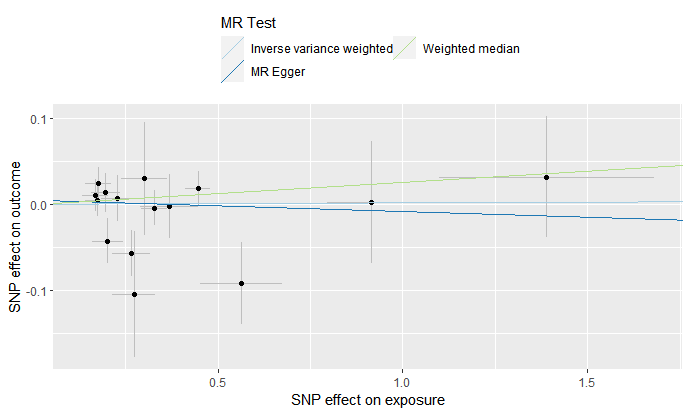 | c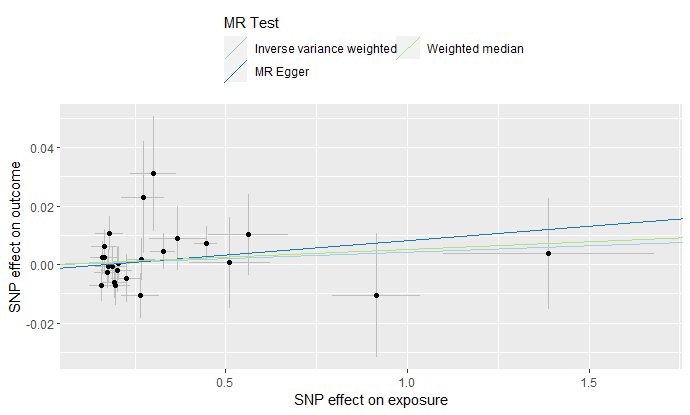 |
| --- | --- | --- |
| d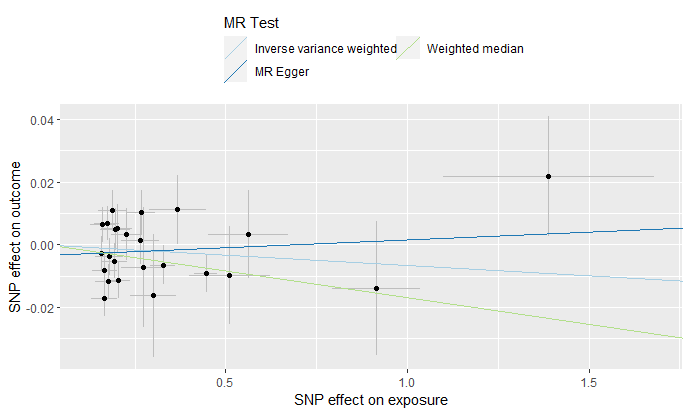 | e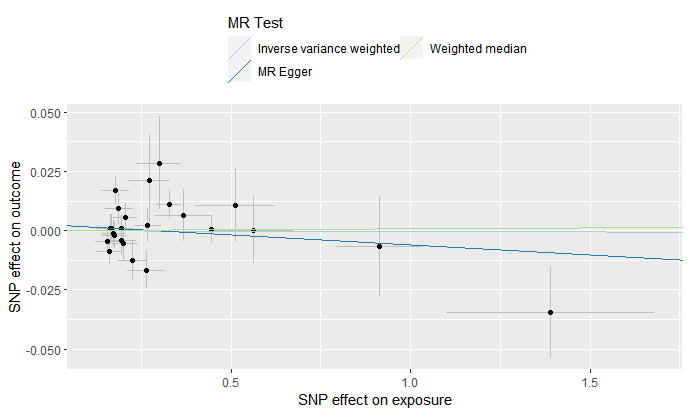 | f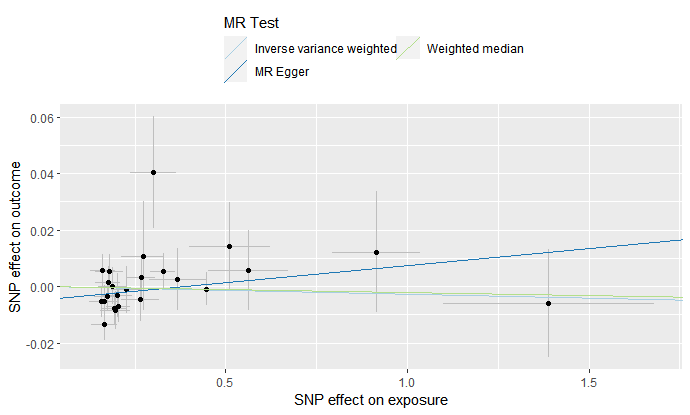 |

**Supplementary Figure. S22** The estimation of pleiotropy for exposure(Malignant neoplasm of thyroid gland) and outcome(Circulating vitamin levels) using MR-Egger intercept. (a) Vitamin A. (b)Vitamin B9. (c)Vitamin B12. (d) Vitamin C. (e)Vitamin D. (f) Vitamin E.

| a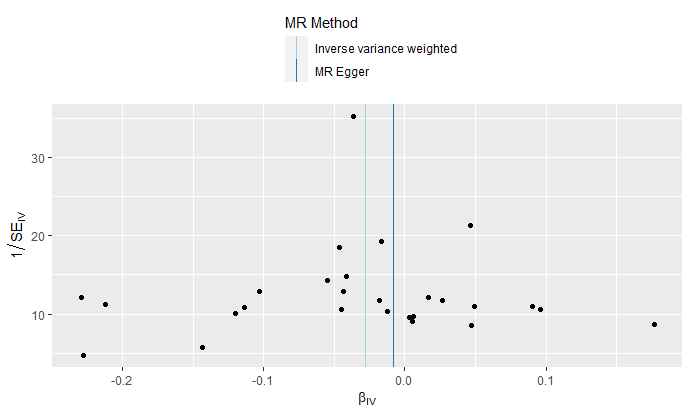 | b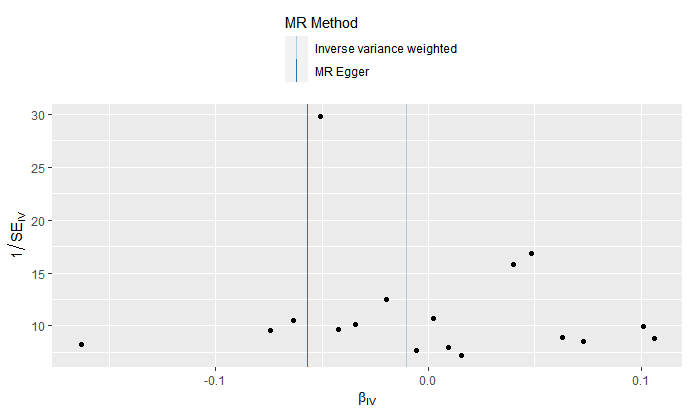 | c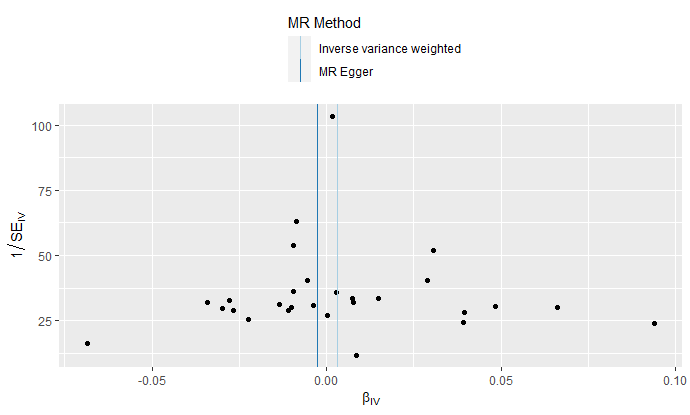 |
| --- | --- | --- |
| d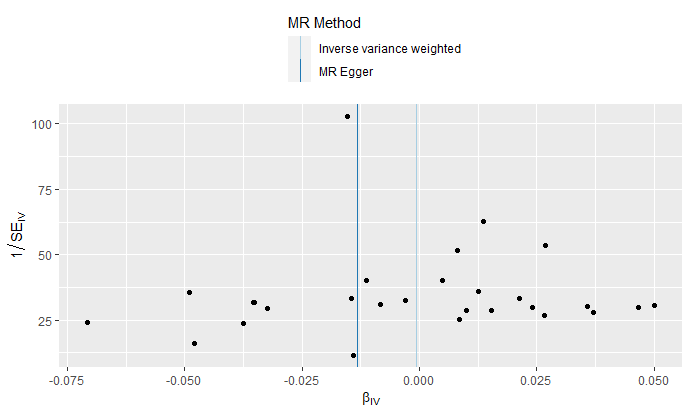 | e | f |

**Supplementary Figure. S23** The estimation of heterogeneity for exposure(Autoimmune hyperthyroidism) and outcome(Circulating vitamin levels) using Funnel plot. (a) Vitamin A. (b)Vitamin B9. (c)Vitamin B12. (d) Vitamin C. (e)Vitamin D. (f) Vitamin E.

| a | b | c |
| --- | --- | --- |
| d | e | f |

**Supplementary Figure. S24** The estimation of heterogeneity for exposure(Autoimmune hypothyroidism) and outcome(Circulating vitamin levels) using Funnel plot. (a) Vitamin A.(b)Vitamin B9. (c)Vitamin B12. (d) Vitamin C. (e)Vitamin D. (f) Vitamin E.

| a | b | c |
| --- | --- | --- |
| d | e | f |

**Supplementary Figure. S25** The estimation of heterogeneity for exposure(Nontoxic goitre/Thyroid nodule) and outcome(Circulating vitamin levels) using Funnel plot. (a) Vitamin A. (b)Vitamin B9. (c)Vitamin B12. (d) Vitamin C. (e)Vitamin D. (f) Vitamin E.

| a | b | c |
| --- | --- | --- |
| d | e | f |

**Supplementary Figure. S26** The estimation of heterogeneity for exposure(Malignant neoplasm of thyroid gland) and outcome(Circulating vitamin levels) using Funnel plot. (a) Vitamin A. (b)Vitamin B9. (c)Vitamin B12. (d) Vitamin C. (e)Vitamin D. (f) Vitamin E.

| a | b | c |
| --- | --- | --- |
| d | e | f |

**Supplementary Figure. S27** MR leave-one-out sensitivity analysis for exposure(Autoimmune hyperthyroidism) on outcome(Circulating vitamin levels). (a) Vitamin A.(b)Vitamin B9. (c)Vitamin B12. (d) Vitamin C. (e)Vitamin D. (f) Vitamin E.

| a | b | c |
| --- | --- | --- |
| d | e | f |

**Supplementary Figure. S28** MR leave-one-out sensitivity analysis for exposure(Autoimmune hypothyroidism) on outcome(Circulating vitamin levels). (a) Vitamin A. (b)Vitamin B9. (c)Vitamin B12. (d) Vitamin C. (e)Vitamin D. (f) Vitamin E.

| a | b | c |
| --- | --- | --- |
| d | e | f |

**Supplementary Figure. S29** MR leave-one-out sensitivity analysis for exposure(Nontoxic goitre/Thyroid nodule) on outcome(Circulating vitamin levels). (a) Vitamin A. (b)Vitamin B9. (c)Vitamin B12. (d) Vitamin C. (e)Vitamin D. (f) Vitamin E.

| a | b | c |
| --- | --- | --- |
| d | e | f |

**Supplementary Figure. S30** MR leave-one-out sensitivity analysis for exposure(Malignant neoplasm of thyroid gland) on outcome(Circulating vitamin levels). (a) Vitamin A.(b)Vitamin B9. (c)Vitamin B12. (d) Vitamin C. (e)Vitamin D. (f) Vitamin E.
